# Coupled changes of bacterial community and function in the gut of mud crab (*Scylla Paramamosain*) in response to Baimang disease

**DOI:** 10.1186/s13568-019-0745-1

**Published:** 2019-02-02

**Authors:** Yiqin Deng, Changhong Cheng, Jiawei Xie, Songlin Liu, Hongling Ma, Juan Feng, Youlu Su, Zhixun Guo

**Affiliations:** 1 Key Laboratory of South China Sea Fishery Resources Exploitation & Utilization, Ministry of Agriculture and Rural Affairs, South China Sea Fisheries Research Institute, Chinese Academy of Fishery Sciences, 309 of Building Keyan, 231 Xingang Xi Road, Guangzhou, China; 2 Key Laboratory of Tropical Marine Bio-resources and Ecology, South China Sea Institute of Oceanology, Chinese Academy of Sciences, Guangzhou, China

**Keywords:** *Scylla paramamosain*, Baimang disease, Intestinal bacterial community, Bacterial function

## Abstract

Increasing evidence has revealed a close association between intestinal bacterial community and hosts health. However, it is unclear whether and what extend Baimang disease alters the intestinal microbiota in mud crab (*Scylla paramamosain*). Here, we conducted intestinal contents Illumina sequencing of healthy and Baimang diseased mud crab (*S. paramamosain*) to understand bacterial community variations among health status. In addition, bacterial functional predication was used to investigate whether and how the bacteria variations further change their functions? The phyla of *Proteobacteria*, *Fusobacteria*, *Cyanobacteria*, *Tenericutes*, *Firmicutes*, *Bacteroidetes*, and *Spirochaetae* constituted over 96.44% of the total intestinal bacteria, with being the dominant taxa. The 7 most significantly different orders, including the increased four orders of *Clostridiales*, *Entomoplasmatales*, *Bacteroidales*, and *Mycoplasmatales* and the decreased three orders of *Vibrionales*, *Campylobacterales*, and *Fusobacteriales*, accounted for 61.14% dissimilarity, probably being the indicator taxa of Baimang disease. Accordingly, 12 Kyoto Encyclopedia of Genes and Genomes orthologies in level 3 shifted significantly at the diseased crabs. Especially, bacterial secretion system, secretion system, lipopolysaccharide biosynthesis proteins and *Vibrio cholerae* pathogenic cycle, being related to bacterial virulence, were reduced. In addition, the reduced butanoate metabolism, and induced methane metabolism and one carbon pool by folate were important metabolic processes of probiotic, such as *Bacteroides* spp. and *Clostridium* spp., with playing critical roles in host health. This study suggests that Baimang disease coupled altered the intestinal bacterial communities and functions, providing timely information for further analysis the influencing mechanism of Baimang disease in mud crab (*S. paramamosain*).

**Electronic supplementary material:**

The online version of this article (10.1186/s13568-019-0745-1) contains supplementary material, which is available to authorized users.

## Introduction

Gut, the most important digestive organ, is colonized by complex symbiont microbiota with multiple functions critical for host health (Ramírez and Romero 2017; Xiong et al. 2018). Numerous studies have shown that gut microbiota affects the nutrient absorption, immune response and disease resistance of the hosts by secreting exogenous enzymes, fixing nitrogen source and producing secondary metabolites, etc., thus profoundly influence the physiology and pathology of hosts (Harris 1993; Bäckhed et al. 2004). For example, Gobet et al. (2018) found that digestive gland bacteria may cooperate to degrade algal polysaccharides to products which are assimilable by the host. The outbreak of diseases probably shapes the composition of intestinal microbial communities (Xiong et al. 2015; Zhu et al. 2016). Shi et al. (2019) found that vibriosis induced marked changes in the gut bacterial composition of crab *Portunus trituberculatus* with driving the gut bacterial community into a kind of diseased status. In addition, the disruption of a normal microbiota would alter bacterial functional composition in the intestine accordingly (Zhu et al. 2016; Zeng et al. 2017; Shi et al. 2019). With the function predication by PICRUSt, Zhu et al. (2016) found that the pathways of focal adhesion and disease infection significantly increased in the diseased shrimp, while the antibacterial pathways decreased accompanied with the variety of intestinal microbial composition. These studies suggest that it is important to evaluate the health status of aquaculture organisms by the changes of intestinal microflora. Furthermore, bacterial functional analysis links the structure and function of intestinal microbial community thus helpful to clarify the pathogenesis.

Mud crab (*Scylla paramamosain*), a commercially important crustacean, is rapidly developed worldwide and widely cultured along the southeast coast of China with salinity around 10 g/L (Meng et al. 2017). Recently, as the rapid expansion of high density and intensive farming, it has suffered from serious diseases caused by bacteria, fungi, parasites, and environmental changes with endangering the sustainable development of mud crab (*S. paramamosain*) industry (Zhu et al. 2018). Baimang disease, a physiological dysfunction in mud crab (*S. paramamosain*), is probably caused by dramatic decreased salinity in aquaculture water, thus plaguing the farmers in southeast China with frequent typhoons and rainstorms (Zheng 2014; Xu et al. 2000). The name of ‘Baimang disease’ comes from the typical symptom of the diseased crab: the muscles inside the pereiopod become milky white and even flow out white mucus while the healthy crab shows blue. The other symptom of Baimang diseased crab showed a frail situation of sluggish action and reducing ingestion (Zheng 2014; Xu et al. 2000). Recent years, studies have focused on the intestinal microbiota in healthy crabs or intestinal bacterial community assembly driving by growth conditions (Li et al. 2007; Chen et al. 2015a), however, little information is available regarding the effect of Baimang disease on the intestinal bacterial community structure and function assembly in mud crab (*S. paramamosain*), thereby shortening our knowledge of underlying the influencing mechanisms of Baimang disease.

Here, we collect the gut contents of healthy and Baimang diseased mud crab (*S. paramamosain*) from 3 crab ponds in Nansha district, Guangzhou, China. Illumina sequencing was conducted to identify the intestinal microbial diversity and to study the bacterial assembly caused by Baimang disease. In addition, bacterial function was predicated with the 16s rDNA gene data by PICRUSt to investigate whether and how the bacterial assembly further change its function? The results will help to reveal the effect of intestinal bacteria on health status of mud crab (*S. paramamosain*), and provide scientific basis for elucidating the influencing mechanism of Baimang disease in mud crab (*S. paramamosain*).

## Materials and methods

### Sample collection

Samples were collected from 3 mud crab (*S. paramamosain*) ponds in Nansha district, Guangzhou, China on November 22th 2017, when Baimang disease broke out with a mortality rate of about 50%. The diseased crabs’ muscles inside the pereiopod became milky white and even flow out white mucus, while the healthy crabs showed blue. Finally, five healthy crabs (H1 to H5) and five diseased crabs (D1 to D5) were collected with an average weight of 291 ± 25 g. The temperature, pH, DO, and salinity values of crab ponds water were 17.37 ± 0.22 °C, 8.43 ± 0.01, 6.66 ± 0.05 mg/L, and 3.92 ± 0.08 g/L (much lower than 10 g/L), respectively. The concentration of NO_3_^−^–N, NO_2_^−^–N, NH_4_^+^–N, PO_4_^3−^P, TDN (total dissolved nitrogen), TDP (total dissolved phosphorus), DOC (dissolved organic carbon) were 0.178 ± 0.040 mg/L, 0.003 mg/L, 0.261 ± 0.032 mg/L, 0.010 mg/L, 0.753 ± 0.141 mg/L, 0.025 ± 0.003 mg/L, and 4.792 ± 0.397 mg/L, respectively. After the measurement of weight, crabs were washed thoroughly using sterile water and disinfected with 75% ethanol for 3–5 min. Crabs were dissected immediately and the whole digestive tracts were removed. The gut contents were collected in sterile tubes and immediately stored at − 80 °C before DNA extraction.

### DNA extraction and Illumina sequencing

The bacterial genomic DNA was extracted using a HiPure Soil DNA Kit B (Magen, China) base on the manufacturer’s instructions. Next generation sequencing library preparations and Illumina MiSeq sequencing were conducted at GENEWIZ, Inc. (Suzhou, China). DNA samples were quantified using a Qubit 2.0 Fluorometer (Invitrogen, Carlsbad, CA, USA). Then V3 and V4 hypervariable regions of prokaryotic 16S rDNA were amplified for generating amplicons and following taxonomy analysis using forward primers containing the sequence “CCTACGGRRBGCASCAGKVRVGAAT” and reverse primers containing the sequence “GGACTACNVGGGTWTCTAATCC”. 1st round PCR products were used as templates for 2nd round amplicon enrichment PCR. Indexed adapters were added to the ends of the 16S rDNA amplicons and DNA libraries were validated by Agilent 2100 Bioanalyzer (Agilent Technologies, Palo Alto, CA, USA), and quantified by Qubit 2.0 Fluorometer. DNA libraries were multiplexed and loaded on an Illumina MiSeq instrument according to manufacturer’s instructions (Illumina, San Diego, CA, USA). Sequencing was performed using a 2 × 300 paired-end (PE) configuration; image analysis and base calling were conducted by the MiSeq Control Software (MCS) embedded in the MiSeq instrument.

### Sequence analysis

The QIIME (1.9.1) data analysis package was used for 16S rDNA gene data analysis (Caporaso et al. 2010). Raw sequences were joined and quality filtered with the default parameters in QIIME. Then the chimeric sequences were detected and removed using UCHIME algorithm. The effective sequences were grouped into operational taxonomic units (OTUs) using the clustering program VSEARCH (1.9.6) (Rognes et al. 2016) against the Silva 119 database pre-clustered at 97% sequence identity. The Ribosomal Database Program (RDP) (Wang et al. 2007) classifier was used to assign taxonomic category to all OTUs at confidence threshold of 0.8. The RDP classifier uses the Silva 123 database (Quast et al. 2012) which has taxonomic categories predicted to the species level. Sequences were rarefied prior to the calculation of alpha and beta diversity statistics. Alpha diversity indexes were calculated in QIIME from rarefied samples using for richness the Ace and Chao1 index, for diversity the Shannon and Simpson indexes. The Bray–Curtis distance matrix was calculated with the OTU table using PRIMER 6 & PERMANOVA + (Clarke and Gorley, 2006).

### Microbial function prediction based on 16S rDNA data

The metagenome functional capacity was inferred with the 16S rDNA sequence data using PICRUSt (version 1.1.0) (Langille et al. 2013). A closed-reference OTU picking was performed on paired-end merged 16S rDNA sequences. The sequences were blasted against the Greengenes database with generating the OTU table. The OTU abundance matrix was firstly normalized to its 16S rDNA copy number. Predicted functional pathways were annotated by using the Kyoto Encyclopedia of Genes and Genomes (KEGG) (Kanehisa et al. 2011) at level 1, level 2 and level 3 KEGG orthology groups (KOs).

### Statistical Analysis

The independent *t* test was conducted to determine the significant difference of alpha diversity indexes, the relative abundance of bacterial community (order level) (Additional file 1: Table S1) and microbial function (KEGG level 3) (Additional file 1: Table S2) (*P *< 0.05 was considered statistically significant). The Bray–Curtis distance matrix (Additional file 1: Tables S3, S4) was used for the bacterial community and function analysis by preliminary one-way permutational multivariate ANOVA (PERMANOVA), principal coordinates analysis (PCoA) and similarity percentage analysis (SIMPER). For healthy and diseased crab groups, the PERMANOVA showed that pond was not a significant effect on bacterial microbiota (Healthy group: Pseudo-F = 1.1679, *P*-perm = 0.405; diseased group: Pseudo-F = 0.4518, *P*-perm = 0.999). Subsequently, a PERMANOVA analysis was ran to examine the effect of Baimang disease on microbial communities and functions with the data of OTU table (Additional file 1: Table S5) and KEGG level 3 table (Additional file 1: Table S2) (*P *< 0.05 was considered statistically significant). Moreover, the PCoA analysis was conducted to investigate the intestinal microbial community differences between healthy and diseased crabs. A SIMPER analysis was used to identify the bacterial taxa driving the differences in diseased crabs. The above-mentioned statistical analyses were performed with PRIMER 6 & PERMANOVA + (Clarke and Gorley, 2006) and IBM SPSS Statistics 19.0 software, respectively.

### Accession number(s)

All the raw reads were deposited in the Sequencing Read Archive (SRA) of NCBI with accession numbers from SRR8357120 to SRR8357129, and Bioproject Number “PRJNA510862”.

## Results

### Bacterial community structure and composition

Quality and chimera filtration of the raw data produced totally 697,738 high quality sequencing reads from 10 samples, with an average of 69,774 reads, ranging from 40,931 to 152,799 (Table 1). By performing the alignment with the effective reads, OTU were pre-clustered at 97% sequence identity. Finally, 268 OTUs were obtained and between 39 and 133 OTUs per sample (mean = 81, n = 10) (Table 1, Additional file 1: Table S5).

**Table 1:** Summary of sequence, OTU, and α-diversity

Samples	No. of reads	No. of OTUs	α-Diversity indices			
			Ace	Chao1	Shannon	Simpson
H1	88,275	133	160.685	164.5	3.635	0.86
H2	48,239	66	52.875	49	2.003	0.617
H3	65,059	66	154.772	148	3.239	0.789
H4	64,532	45	94.259	83.75	2.05	0.659
H5	62,431	121	109.001	107	3.156	0.811
D1	40,931	60	42.705	40.667	1.521	0.557
D2	50,220	88	74.62	73	3.426	0.862
D3	51,725	39	234.746	196.565	2.677	0.738
D4	88,275	62	160.685	164.5	3.635	0.86
D5	152,799	132	100.413	93.6	1.122	0.414

**Fig. 1:**
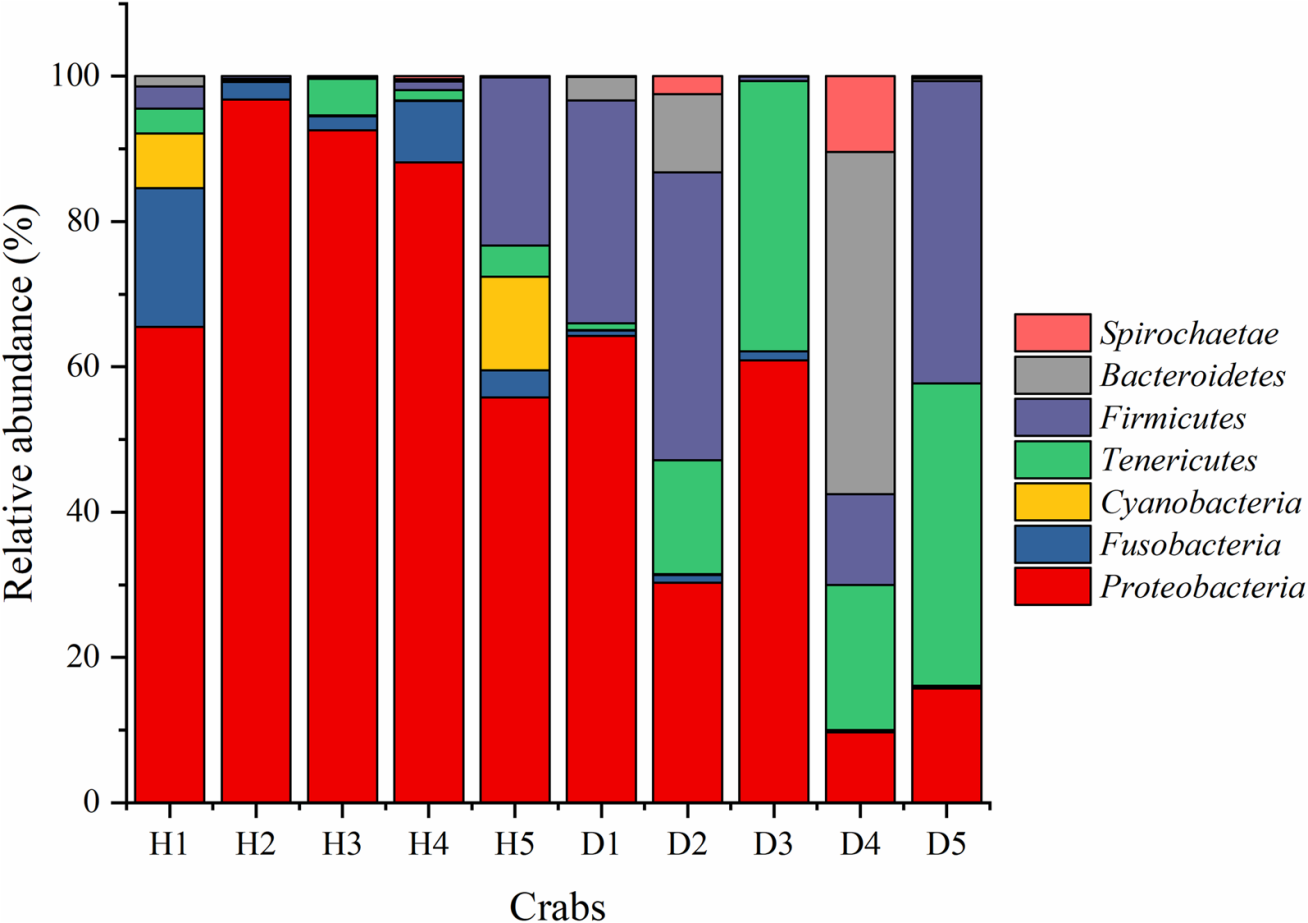
Relative abundance of the dominant (relative abundance > 5% at least in one sample) phyla within different samples

The OTUs have been classified into 15 phyla and sequences that could not be classified into any known groups were assigned as ‘unclassified’ (Additional file 1: Table S6). The dominant (relative abundance > 5% at least in one sample) phyla were *Proteobacteria*, *Fusobacteria*, *Cyanobacteria*, *Tenericutes*, *Firmicutes*, *Bacteroidetes*, and *Spirochaetae* (Fig. 1). In total, these dominant phyla accounted for over 96.44% of the bacterial sequences. *Proteobacteria* was the most abundant phylum among the 5 healthy samples, while only in 2 (D1 and D3) diseased samples (Additional file 1: Table S6, Fig. 1). Additionally, only in D4 sample, the *Spirochaetae* constituted over 5% (10.36%) (Additional file 1: Table S6, Fig. 1).

**Fig. 2:**
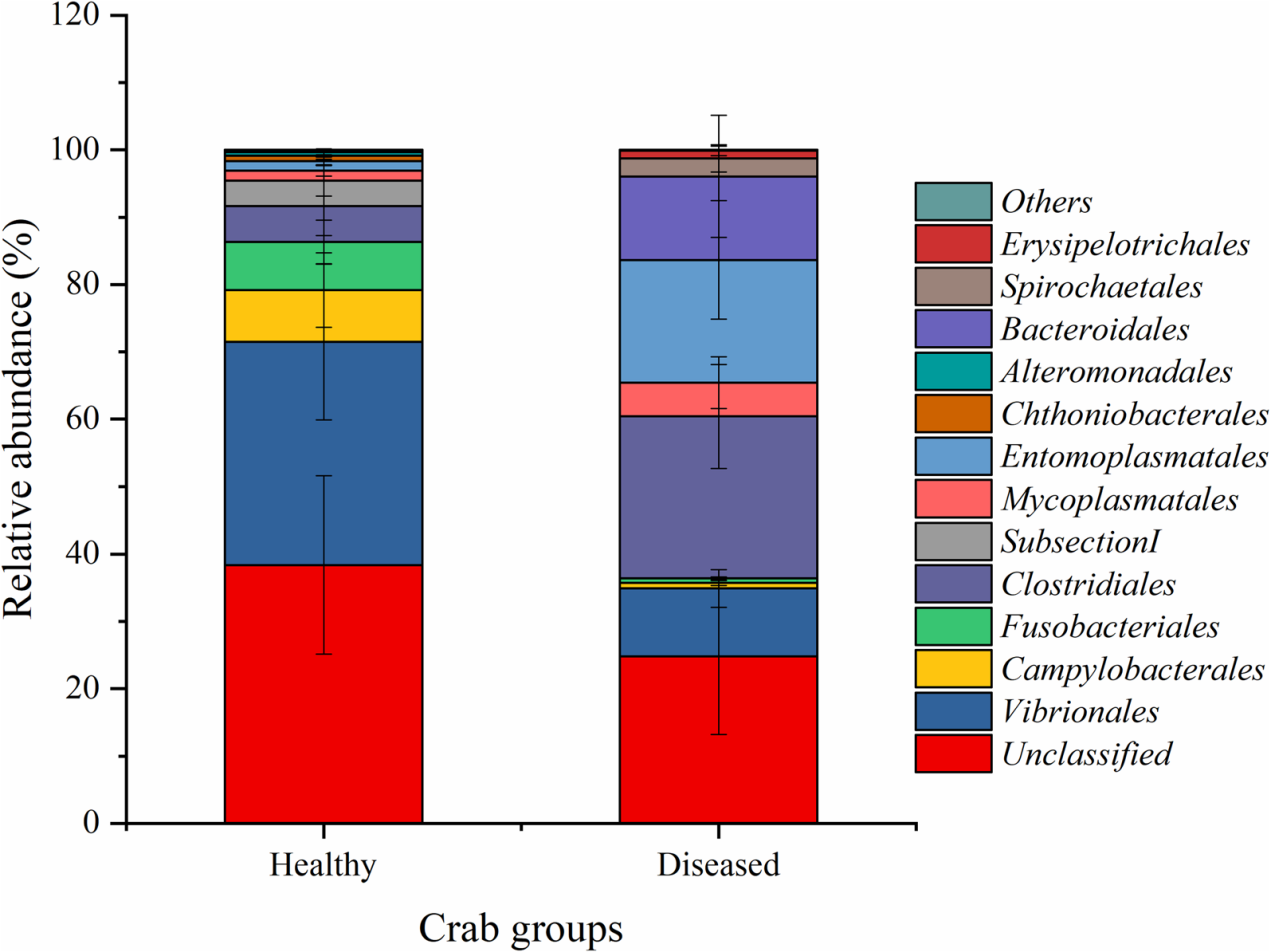
Microbial community composition of the top 10 most abundant orders averaged over each crab group. Values show means and 1 standard error of mean (mean ± SEM, n = 5)

At order level, a total of 39 taxa were identified. The top 10 most abundant orders were *Vibrionales*, *Mycoasmatales*, *Campylobacterales*, *Fusobacteriales*, *Clostridiales*, *SubsectionI*, *Erysipelotrichales*, *Entomoplasmatales*, *Chthoniobacterales*, *Bcteroidales*, *Alteromonadales*, and *Spirochaetales*, respectively, which accounted for 98.24% and 99.08% of the total intestinal bacteria in healthy and diseased crabs, respectively. (Figure 2, Additional file 1: Fig. S1, and Table S1).

### Variation in bacterial community structure across health status

**Fig. 3:**
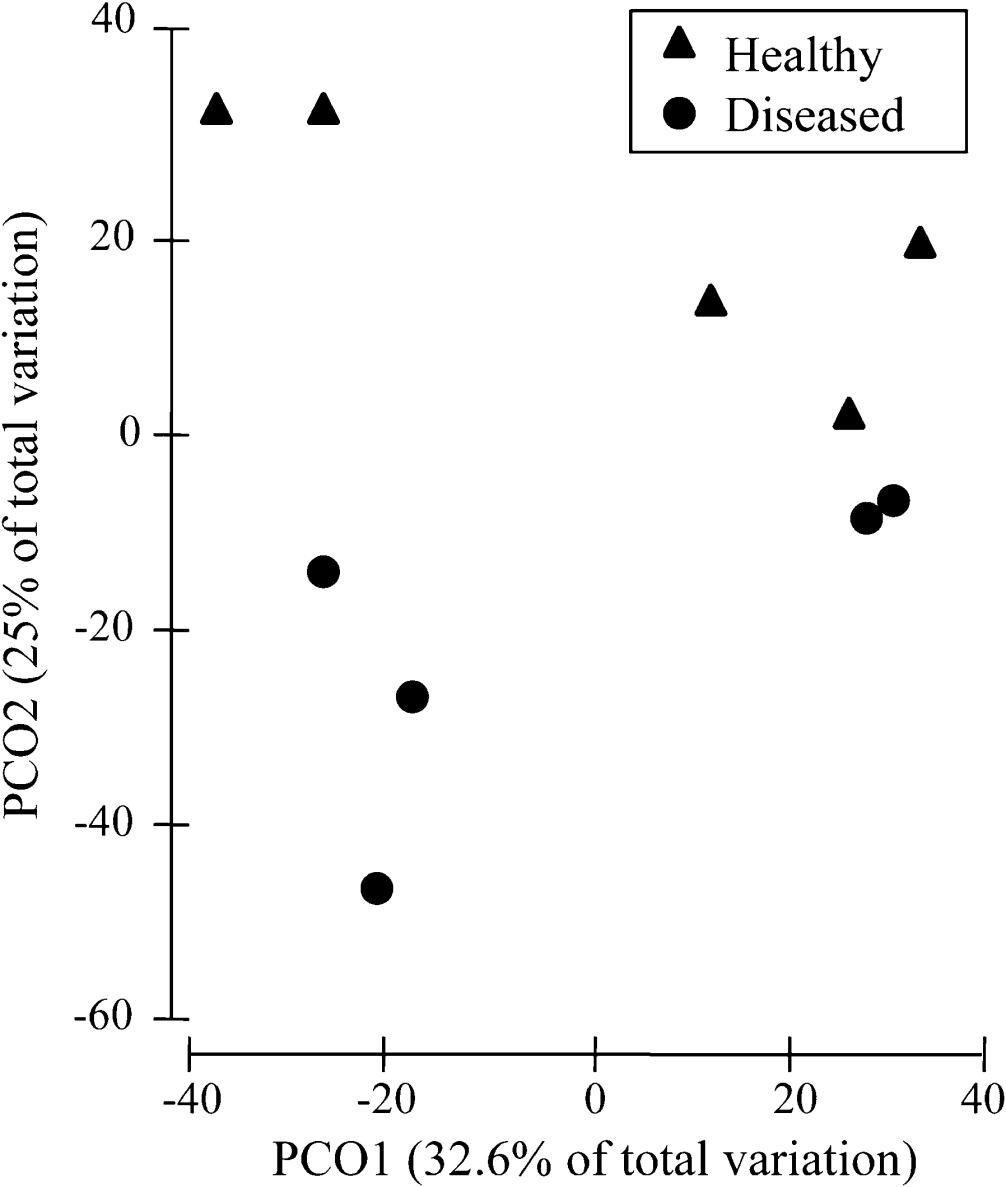
PCoA analysis for the dissimilarity (Bray–Curtis distance) intestinal bacterial community composition of mud crab (*S. paramamosain*) associated with Baimang disease

The α-diversity indices showed similar intestinal bacterial richness (Ace, Chao1) and diversity (Shannon, Simpson) between the healthy and diseased crabs (independent *t* test,F = 1.801, 1.221, 1.736, and 3.480; *P *= 0.839, 0.931, 0.587, and 0.557) (Table 1). PERMANOVA analysis indicated that the effect of Baimang disease on the intestinal bacterial communities was marginally significant (Pseudo-F = 1.8828, *P*-perm = 0.064) and the order communities of *Vibrionales* and *Clostridiales* was marginally significant (independent-samples *t* test, F = 1.918 and 2.116; *P *= 0.091 and 0.067). In addition, the Axis 2 (PCO2) of PCoA analysis separated the healthy group from the diseased group (Fig. 3). First two axes (PCO1 and PCO2) explained 32.6% and 25% of the composition variation, respectively (Fig. 3). SIMPER analysis showed that the 7 most significantly different orders accounted for 61.14% dissimilarity of the two groups (Table 2). The contribution of *Clostridiales*, *Entomoplasmatales*, and *Vibrionales* were all over 10% (Table 2). The abundance of *Clostridiales*, *Entomoplasmatales*, *Bacteroidales*, and *Mycoplasmatales* were increased in the diseased group, while the abundance of *Vibrionales*, *Campylobacterales*, and *Fusobacteriales* were decreased (Table 2).

**Table 2:** Results of the order-level SIMPER analysis giving the dissimilarities (73.04%) of total bacterial communities between groups (the data was square root transformed)

Orders	Average abundance of group H	Average abundance of group D	Average Dissimilarity	Contribution (%)	Cumulative contribution (%)
*Clostridiales*	1.61	4.40	6.73	12.90	12.90
*Entomoplasmatales*	0.98	3.29	6.16	11.82	24.72
*Vibrionales*	5.42	3.05	5.36	10.29	35.01
*Bacteroidales*	0.18	2.50	4.71	9.03	44.04
*Campylobacterales*	1.98	0.78	3.27	6.27	50.31
*Fusobacteriales*	2.41	0.76	3.21	6.15	56.46
*Mycoplasmatales*	1.04	1.68	2.44	4.68	61.14

Group H is the healthy crab group, and group D is the Baimang diseased crab group

### Function predication and the difference induced by Baimang disease

**Fig. 4:**
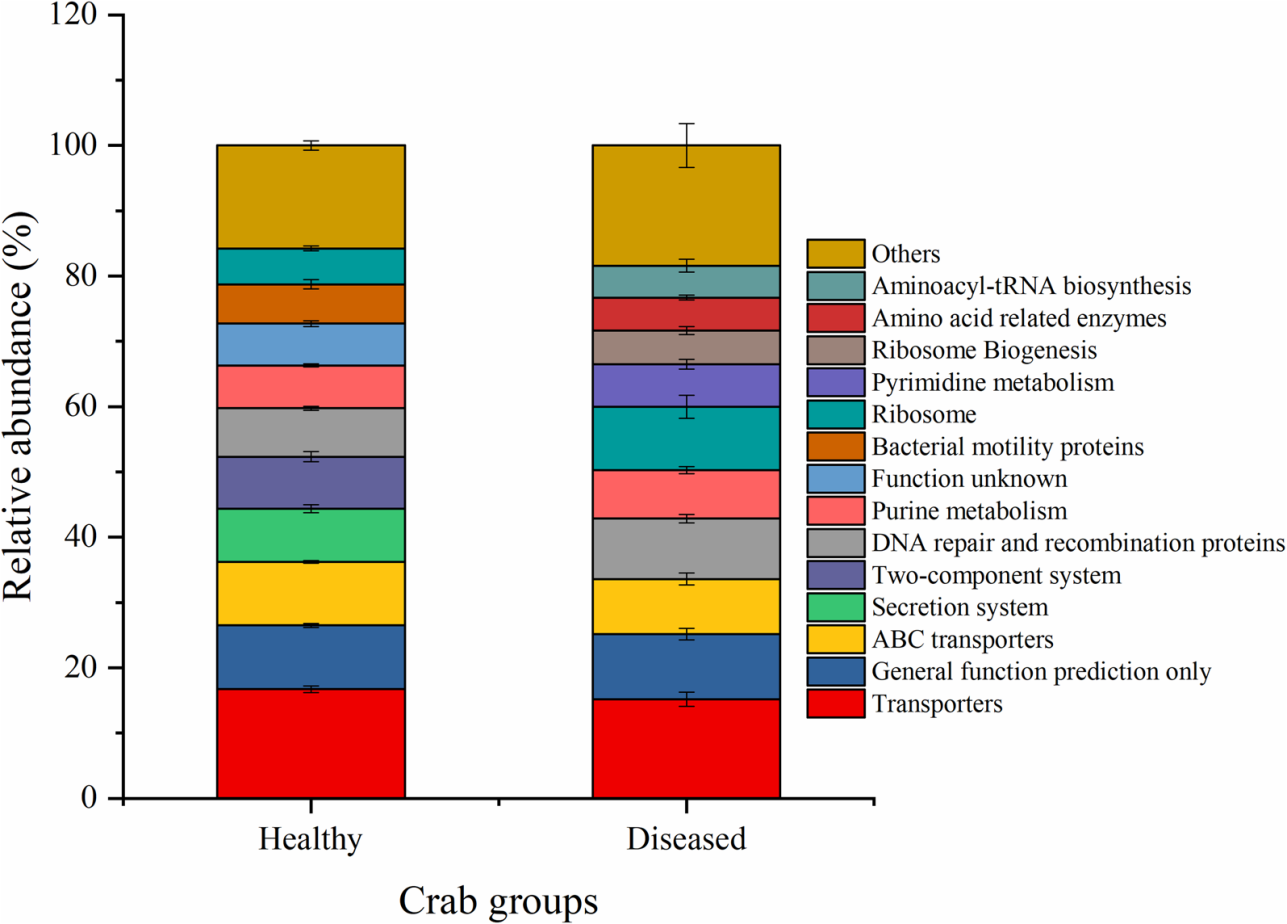
Microbial functions of the top 10 most abundant KEGG level 3 categories averaged over each crab group. Values show means and 1 standard error of mean (mean ± SEM, n = 5)

A total of 246 KEGG pathways were generated using the OTU table data of the gut microbiota based on PICRUSt (Additional file 1: Table S2). The top 10 most abundant KEGG level 3 categories showed that the intestinal microbiota was enriched with functions relating to transporters, ABC transporters, secretion system, two-component system, DNA repair and recombination proteins, purine metabolism, bacterial motility proteins, ribosome, pyrimidine metabolism, ribosome Biogenesis, amino acid related enzymes, and aminoacyl-tRNA biosynthesis (Fig. 4, Additional file 1: Fig. S2, and Table S2).

**Fig. 5:**
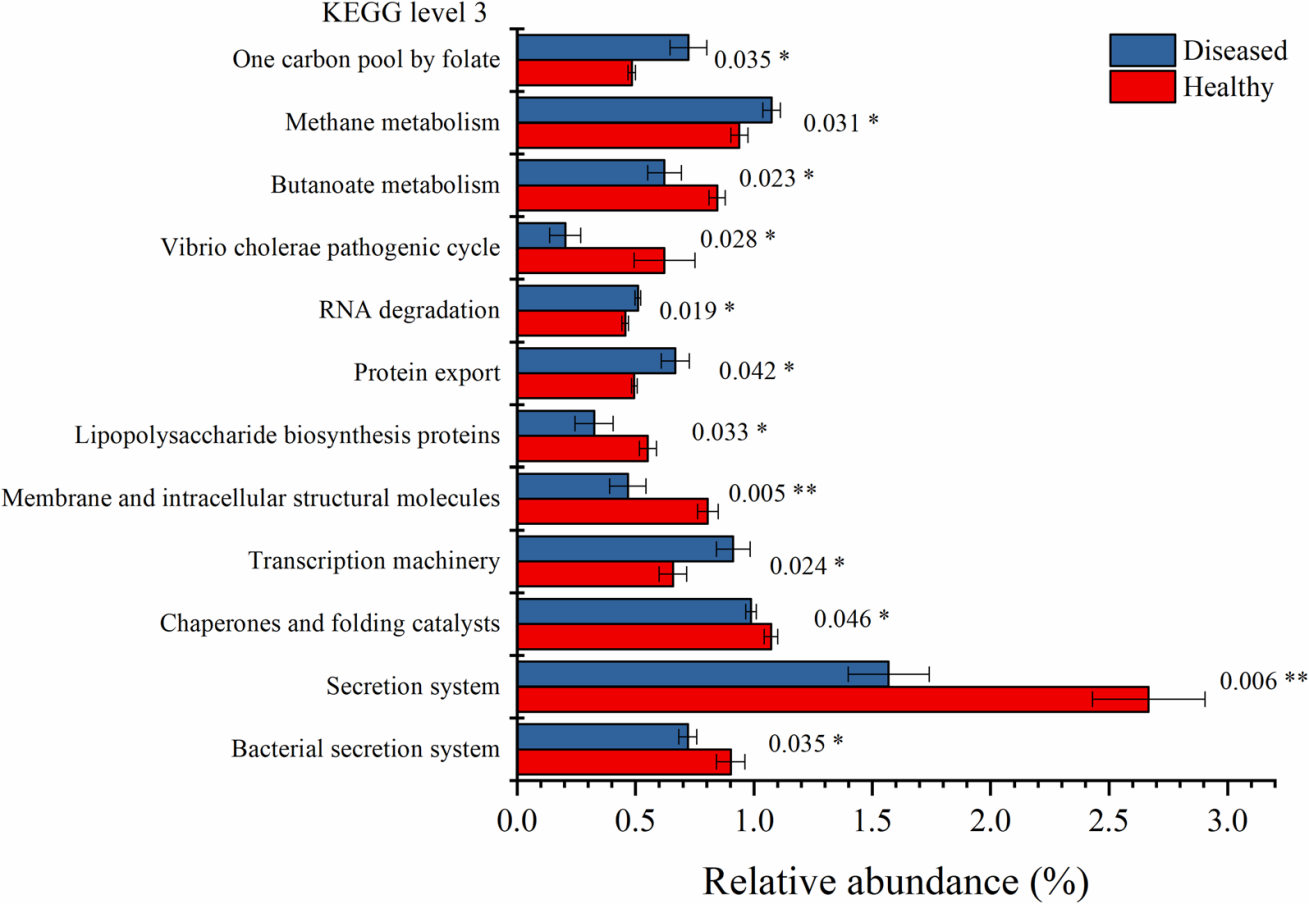
Predicted functions of the intestinal microbiota that varies significantly at between the healthy and diseased crabs (*P < 0.05, **P < 0.01)

PERMANOVA analysis showed that the effect of Baimang disease on the microbial functions was significant (Pseudo-F = 3.0177, *P*-perm = 0.015). Moreover, independent-samples *t* test showed that there were totally 12 KOs (KEGG level3) shift significantly in the diseased crabs with the relative abundance of at least one group was over than 0.5%. Seven of them were significantly reduced with involving into bacterial secretion system, secretion system, chaperones and folding catalysts, membrane and intracellular structural molecules, lipopolysaccharide biosynthesis proteins, *Vibrio cholerae* pathogenic cycle, and butanoate metabolism (Fig. 5 and Additional file 1: Table S7). However, the KOs of transcription machinery, protein export, RNA degradation, methane metabolism, and one carbon pool by folate were significantly induced in the diseased intestinal microbiota (Fig. 5 and Additional file 1: Table S7).

## Discussion

The epidemic in mud crab farms named as ‘Baimang disease’, which breaks out mainly after rainstorms as the salinity of aquaculture water drop sharply, results in large economic losses in crab farming in southeast China. Numerous host diseases has been linked to the disruption of intestinal microbial communities (Xiong et al. 2015; Zhu et al. 2016; Xiong et al. 2018); therefore, unraveling the bacterial community assembly could help to elucidate the pathogenesis and guide the establishment of new strategies against diseases (Matsuyama et al. 2017; Xiong et al. 2017). However, there is little information about how and to what extent the bacterial intestinal community is altered by the occurrence of disease especially by Baimang disease in mud crab (*S. paramamosain*) (Li et al. 2012) limiting our understanding of its pathogenesis and the options of its treatment. Here we used the 16S rDNA Illumina sequencing method to study the effects of Baimang disease on the bacterial community assembly in mud crab (*S. paramamosain*). Further, the bacterial function was predicated to investigate whether and how the bacteria assembly further alters its function?

The results indicated that *Proteobacteria*, *Fusobacteria*, *Cyanobacteria*, *Tenericutes*, *Firmicutes*, and *Bacteroidetes* were the dominant intestinal phyla among the healthy samples, consistent with the previous study (Li et al. 2012). There was no difference in alpha diversity between healthy and diseased groups. However, PCoA analysis indicated that Baimang disease alter the Beta diversity. These results suggested that Baimang disease changes the intestinal bacteria composition but not the bacteria richness or diversity.

SIMPER analysis showed that the 7 most significantly different orders accounted for 61.14% dissimilarity of the two groups, probably being the indicator taxa of Baimang disease. Among them, the abundance of *Clostridiales*, *Entomoplasmatales*, *Bacteroidales*, and *Mycoplasmatales* belonging to the phyla of *Firmicutes*, *Bacteroidetes* and *Tenericutes* were increased in the diseased group, while the abundance of *Vibrionales*, *Campylobacterales*, and *Fusobacteriales* belonging to the phyla of *Proteobacteria* and *Fusobacteria* were decreased. Zhang et al. (2016) found that *Proteobacteria* and *Fusobacteria* were decreased in lower salinity group of Nile tilapia (fish reared in fresh water), and *Firmicutes* and *Tenericutes* were more abundant while *Bacteroidales* was less abundant in lower salinity group of Pacific white shrimp. Similarly, the dropped salinity resulting into Baimang disease of mud crabs may lead to the decrease of *Proteobacteria* and *Fusobacteria* and increase of *Firmicutes* and *Tenericutes* which probably involved into salinity stress and participate in the osmotic pressure regulation. For example, Harris (1993) reported that the *Vibrio* (belonging to *Proteobacteria*) attached to the hindgut wall may play a role in ion transport across the gut wall, and thus contribute to osmotic regulation. However, the opposite changing trend of *Bacteroidales* needs further study, especially the interaction study of host-bacteria. In addition, many species of *Clostridiales* were reported to sporulate, generating dormant and resistant spores that can survive in terrible environment, such as the absence of nutrients due to host diseases (Paredes-Sabja et al. 2011). Therefore, the occurrence of Baimang disease led to the imbalance of intestinal microorganisms, thus probably increase the relative abundance of *Clostridiales* which had strong environmental adaptability.

Function predication indicated that the KOs of bacterial secretion system, secretion system, chaperones and folding catalysts, membrane and intracellular structural molecules, lipopolysaccharide biosynthesis proteins, *Vibrio cholerae* pathogenic cycle, and butanoate metabolism were reduced, while the KOs of transcription machinery, protein export, RNA degradation, methane metabolism, and one carbon pool by folate were significantly induced in the diseased group. Among them, the reduced KOs of bacterial secretion system, secretion system, lipopolysaccharide biosynthesis proteins and *Vibrio cholerae* pathogenic cycle are related to bacterial virulence (Pier 2007; Green and Mecsas 2016). Accordingly, *Vibrionales* and *Campylobacterales*, several species of which, such as *Vibrio* spp., *Arcobacter* spp. and *Campylobacter* spp., were putative pathogens with threatening host health (Eppinger et al. 2004; Vandenberg et al. 2004; Wang 2011; Liu et al. 2018), were decreased in the diseased group. Folate, the coenzyme of one-carbon units transferase system, can be synthesized in probiotic bacteria, such as *Bacteroides* spp. (belonging to *Bacteroidales*), and assigned to be important for host health (Poh et al. 2018). Methane metabolism is always happened in anaerobic bacteria, including the species of *Bacteroides* spp. and *Clostridium* spp., and reported to affect the gastrointestinal neuromuscular function of hosts (Mathur et al. 2016). The increase of *Clostridiales* and *Bacteroidales* probably contribute to the induction of methane metabolism and one carbon pool by folate. Moreover, butanoate metabolism is an important metabolic process of probiotic *Clostridium* spp. (belonging to *Clostridiales*), playing a critical role in the prevention and treatment of intestinal diseases, such as enteritis and intestinal tumorigenesis (Chen et al. 2015b). The more abundant of *Clostridiales* while the reduction of butanoate metabolism should be further researched. Overall, dysbiosis of intestine in mud crab (*S. paramamosain*) leads to the disorder of their physiological functions.

In cloclusion, both the intestinal microbiota and bacterial functions altered significantly due to Baimang disease. The results are helpful to reveal the response mechanism of intestinal microflora to the Baimang disease in mud crab (*S. paramamosain*) with providing guidance for evaluate host health status and laying scientific basis for elucidate the infulencing mechanism of Baimang disease. In the furture study, we recommend to isolate intestinal probiotics, such as *Clostridium* spp. and *Bacteroides* spp., and investgate their resistance mechinism to Baimang disease, thus develope defense measures against Baimang diseases.

## Additional file

**Additional file 1.** Additional figures and tables.
