# Supplementary material for: Coupled changes of bacterial community and function in the gut of mud crab (Scylla Paramamosain) in response to Baimang disease
Source: AMB Express. 2019 Feb 2;9:18. doi: 10.1186/s13568-019-0745-1 (PMC6359999; doi:10.1186/s13568-019-0745-1)
Supplement: Supplementary file 1 — Additional file 1. Additional figures and tables. [file 13568_2019_745_MOESM1_ESM.pdf]

AMB Express

Coupled changes of bacterial community and function in the gut of mud crab (*Scylla Paramamosain*) in response to Baimang disease

Yiqin Deng[1], Changhong Cheng[1], Jiawei Xie[1], Songlin Liu[2], Hongling Ma[1], Juan Feng[1], Youlu Su[1], Zhixun Guo[1*]

[1] Key Laboratory of South China Sea Fishery Resources Exploitation & Utilization, Ministry of Agriculture and Rural Affairs, South China Sea Fisheries Research Institute, Chinese Academy of Fishery Sciences, Guangzhou, China

[2] Key Laboratory of Tropical Marine Bio-resources and Ecology, South China Sea Institute of Oceanology, Chinese Academy of Sciences, Guangzhou, China

**Corresponding author:*

309 of Building Keyan

231 Xingang Xi Road, South China Sea Fisheries Research Institute

Guangzhou, China

Tel: +86-020-89108321

Email: guozhixun1@163.com

Table S1 Relative abundance (%) of microbial community (order level)

Taxon	H1	H2	H3	H4	H5	D1	D2	D3	D4	D5
Entomoplasmatales	1.96	0.01	0.24	1.13	3.37	0.13	13.27	37.14	0.00	40.23
Clostridiales	2.26	0.32	0.04	1.14	22.25	30.60	39.23	0.54	11.02	37.74
Vibrionales	29.63	23.56	76.99	24.05	9.69	14.07	18.96	4.13	6.31	6.65
Unclassified	5.13	73.18	11.16	58.88	41.56	49.06	10.37	56.61	1.53	6.31
Erysipelotrichales	0.68	0.00	0.20	0.03	0.05	0.06	0.34	0.04	1.41	3.74
Campylobacterales	28.68	0.00	3.82	4.96	0.14	1.04	0.31	0.11	0.28	2.18
Mycoplasmatales	1.35	0.12	4.82	0.34	0.80	0.78	2.40	0.11	19.90	1.33
Spirochaetales	0.00	0.00	0.00	0.39	0.00	0.10	2.43	0.00	10.36	0.29
Rickettsiales	0.01	0.00	0.38	0.22	0.03	0.03	0.13	0.06	0.00	0.23
SubsectionI	7.07	0.22	0.09	0.02	10.80	0.04	0.07	0.01	0.00	0.23
Desulfovibrionales	0.01	0.00	0.00	0.00	0.00	0.01	0.34	0.00	2.03	0.21
Pseudomonadales	0.01	0.00	0.00	0.01	0.16	0.02	0.03	0.01	0.00	0.20
Bacteroidales	0.05	0.00	0.02	0.30	0.00	3.23	10.61	0.04	46.85	0.17
Flavobacteriales	1.26	0.00	0.00	0.02	0.15	0.01	0.02	0.00	0.00	0.14
Fusobacteriales	18.55	2.39	1.96	8.49	3.59	0.79	1.06	1.20	0.29	0.05
Frankiales	0.00	0.00	0.00	0.00	0.01	0.00	0.00	0.00	0.00	0.04
SAR11_clade	0.00	0.00	0.00	0.00	0.00	0.00	0.00	0.00	0.00	0.03
Chthoniobacterales	1.32	0.05	0.01	0.00	2.63	0.02	0.06	0.00	0.00	0.02
Oligoflexales	0.76	0.03	0.00	0.00	1.28	0.00	0.02	0.00	0.00	0.02
Burkholderiales	0.07	0.01	0.04	0.00	0.02	0.00	0.01	0.00	0.00	0.02
Acidimicrobiales	0.17	0.01	0.01	0.00	0.27	0.00	0.00	0.00	0.00	0.02
PeM15	0.09	0.00	0.00	0.00	0.13	0.00	0.00	0.00	0.00	0.02
Sphingobacteriales	0.00	0.00	0.01	0.00	0.00	0.00	0.00	0.00	0.00	0.02
Alteromonadales	0.39	0.07	0.10	0.01	1.85	0.00	0.24	0.00	0.00	0.01

Xanthomonadales	0.24	0.03	0.04	0.00	0.01	0.00	0.01	0.00	0.00	0.01
Rhodobacterales	0.03	0.00	0.00	0.00	0.01	0.00	0.01	0.00	0.00	0.01
Legionellales	0.11	0.00	0.00	0.00	0.42	0.00	0.00	0.00	0.00	0.01
Solirubrobacterales	0.01	0.00	0.00	0.00	0.05	0.00	0.00	0.00	0.00	0.01
SAR324_clade (Marine_group_B)	0.01	0.00	0.00	0.00	0.01	0.00	0.00	0.00	0.00	0.01
Chlorobiales	0.00	0.00	0.00	0.00	0.00	0.00	0.00	0.00	0.00	0.01
SubsectionIII	0.00	0.00	0.00	0.00	0.00	0.00	0.00	0.00	0.00	0.01
Methylophilales	0.00	0.00	0.00	0.00	0.00	0.00	0.00	0.00	0.00	0.01
Myxococcales	0.02	0.00	0.00	0.00	0.00	0.00	0.00	0.00	0.01	0.00
Enterobacteriales	0.05	0.00	0.00	0.00	0.63	0.00	0.04	0.00	0.00	0.00
Corynebacteriales	0.02	0.00	0.00	0.00	0.07	0.00	0.00	0.00	0.00	0.00
Ambiguous_taxa	0.01	0.00	0.01	0.00	0.01	0.00	0.00	0.00	0.00	0.00
SubsectionIV	0.00	0.00	0.00	0.00	0.01	0.00	0.00	0.00	0.00	0.00
Gaiellales	0.00	0.00	0.00	0.00	0.01	0.00	0.00	0.00	0.00	0.00
Desulfobacterales	0.01	0.00	0.03	0.00	0.00	0.00	0.00	0.00	0.00	0.00
Subgroup_10	0.02	0.00	0.00	0.00	0.00	0.00	0.00	0.00	0.00	0.00

Table S2 Relative abundance (%) of microbial function (KEGG level 3)

CategoryL3	H1	H2	H3	H4	H5	D1	D2	D3	D4	D5
Transporters	5.41	5.38	5.19	5.81	5.46	5.36	6.17	4.66	4.45	5.18
General function prediction only	3.31	3.13	3.09	3.01	3.38	3.24	3.36	3.21	3.80	3.13
ABC transporters	3.01	3.24	3.16	3.33	3.12	3.54	3.29	2.12	2.40	2.96
DNA repair and recombination proteins	2.51	2.21	2.32	2.49	2.55	2.58	2.81	4.50	2.56	3.60
Ribosome	2.02	1.47	1.65	1.85	1.96	1.99	2.58	6.23	2.13	4.21
Purine metabolism	2.25	1.98	2.06	2.30	2.11	2.05	2.23	3.67	2.07	2.86
Two-component system	2.23	3.39	3.28	1.95	2.24	2.54	1.98	0.42	2.12	1.28

Secretion system	2.15	3.07	3.18	2.89	2.04	2.17	1.70	1.25	1.26	1.46
Pyrimidine metabolism	1.67	1.35	1.44	1.68	1.63	1.60	1.89	3.57	1.71	2.59
Bacterial motility proteins	1.59	2.62	2.54	1.30	1.82	2.70	1.72	0.22	1.48	1.42
Function unknown	1.86	2.56	2.48	1.71	1.84	1.80	1.45	1.07	1.35	1.23
Ribosome Biogenesis	1.41	1.44	1.51	1.44	1.40	1.41	1.46	2.85	1.21	2.07
Peptidases	1.56	1.46	1.47	1.61	1.60	1.70	1.55	1.28	1.72	1.48
Amino acid related enzymes	1.32	1.14	1.20	1.28	1.31	1.40	1.48	2.49	1.41	1.97
Chromosome	1.31	1.25	1.31	1.48	1.37	1.40	1.54	2.06	1.32	1.78
Transcription factors	1.39	1.92	1.81	1.41	1.60	1.63	1.50	0.45	1.40	0.94
Aminoacyl-tRNA biosynthesis	1.03	0.68	0.77	0.98	0.98	1.03	1.29	3.24	0.94	2.21
DNA replication proteins	0.99	0.86	0.95	0.96	1.02	0.98	1.35	2.89	1.07	1.98
Other ion-coupled transporters	1.30	1.55	1.58	1.64	1.27	1.36	1.15	0.37	1.48	0.71
Glycolysis / Gluconeogenesis	1.12	0.90	0.91	1.12	1.01	0.96	1.17	2.34	0.91	1.66
Amino sugar and nucleotide sugar metabolism	1.24	1.19	1.10	1.22	1.12	1.02	1.24	1.10	1.26	0.99
Oxidative phosphorylation	1.20	0.84	0.90	1.09	1.28	1.00	1.10	1.31	1.08	1.29
Arginine and proline metabolism	1.12	1.07	1.03	1.01	1.21	1.32	1.08	0.73	1.25	1.04
Pyruvate metabolism	1.06	1.07	1.05	1.01	1.04	1.08	1.08	1.18	0.99	1.15
Chaperones and folding catalysts	1.04	1.13	1.15	1.00	1.04	0.97	0.94	1.07	0.98	0.97
Methane metabolism	0.94	0.84	0.88	1.03	1.01	1.18	1.03	0.96	1.10	1.10
Carbon fixation pathways in prokaryotes	0.98	0.98	1.00	1.06	0.96	1.21	0.89	0.74	1.13	0.96
Others	0.96	0.65	0.71	1.02	1.01	0.86	1.03	1.19	1.19	1.03
Homologous recombination	0.82	0.73	0.76	0.73	0.80	0.88	0.94	1.71	0.90	1.30
Porphyrin and chlorophyll metabolism	1.19	0.83	0.76	1.01	1.17	1.20	1.00	0.38	1.18	0.84
Translation proteins	0.94	0.86	0.86	0.87	0.94	0.91	0.88	1.28	0.81	1.08
Bacterial chemotaxis	0.80	1.47	1.40	0.53	0.83	1.37	0.89	0.09	1.06	0.65
Alanine, aspartate and glutamate metabolism	0.97	0.91	0.90	1.02	0.96	0.94	0.97	0.50	1.13	0.72

Energy metabolism	0.71	0.80	0.84	0.92	0.83	1.01	0.84	0.60	1.03	0.79
Cysteine and methionine metabolism	0.84	0.79	0.76	0.81	0.88	1.00	0.83	0.58	0.85	0.79
Glycine, serine and threonine metabolism	0.83	0.94	0.91	0.80	0.87	0.95	0.80	0.35	1.02	0.65
Bacterial secretion system	0.82	0.90	0.97	1.09	0.74	0.66	0.73	0.85	0.63	0.73
Pentose phosphate pathway	0.79	0.62	0.61	0.79	0.78	0.65	0.87	1.26	0.78	0.95
Transcription machinery	0.69	0.56	0.58	0.59	0.87	0.67	0.89	1.12	0.95	0.93
Mismatch repair	0.68	0.61	0.64	0.66	0.68	0.73	0.80	1.25	0.76	1.01
Butanoate metabolism	0.76	0.85	0.85	0.96	0.80	0.82	0.65	0.38	0.61	0.65
Fructose and mannose metabolism	0.84	0.69	0.60	0.79	0.66	0.55	0.74	0.91	0.84	0.67
DNA replication	0.59	0.51	0.55	0.52	0.56	0.55	0.72	1.57	0.63	1.08
Replication, recombination and repair proteins	0.73	0.87	0.82	0.66	0.76	0.74	0.63	0.67	0.67	0.66
Protein folding and associated processing	0.85	0.72	0.71	0.78	0.81	0.66	0.63	0.46	0.85	0.55
Nitrogen metabolism	0.69	0.69	0.70	0.73	0.72	0.66	0.77	0.34	1.03	0.49
Starch and sucrose metabolism	0.63	0.55	0.49	0.55	0.68	0.58	0.87	0.77	1.10	0.62
Citrate cycle (TCA cycle)	0.70	0.67	0.67	0.66	0.57	0.82	0.55	0.56	0.81	0.68
Valine, leucine and isoleucine biosynthesis	0.67	0.56	0.56	0.61	0.67	0.61	0.68	0.66	0.72	0.67
Flagellar assembly	0.53	0.87	0.89	0.55	0.65	1.04	0.75	0.09	0.39	0.64
Membrane and intracellular structural molecules	0.73	0.90	0.91	0.80	0.69	0.58	0.51	0.24	0.65	0.35
Glycerophospholipid metabolism	0.52	0.51	0.55	0.55	0.53	0.53	0.60	1.23	0.46	0.87
Phosphotransferase system (PTS)	0.81	0.85	0.72	0.95	0.45	0.34	0.37	1.05	0.18	0.53
Propanoate metabolism	0.60	0.65	0.67	0.73	0.62	0.67	0.61	0.46	0.58	0.64
Carbon fixation in photosynthetic organisms	0.61	0.48	0.48	0.58	0.54	0.56	0.65	1.00	0.53	0.80
Phenylalanine, tyrosine and tryptophan biosynthesis	0.71	0.62	0.59	0.67	0.67	0.87	0.64	0.10	0.77	0.49
One carbon pool by folate	0.53	0.46	0.46	0.46	0.52	0.63	0.58	0.99	0.61	0.81
Protein export	0.54	0.48	0.49	0.47	0.50	0.56	0.59	0.87	0.59	0.73
Translation factors	0.49	0.41	0.43	0.45	0.46	0.50	0.55	1.10	0.51	0.80

Peptidoglycan biosynthesis	0.63	0.56	0.55	0.64	0.67	0.62	0.69	0.10	0.78	0.38
Photosynthesis proteins	0.91	0.32	0.30	0.40	0.94	0.32	0.43	0.98	0.32	0.69
Lipid biosynthesis proteins	0.59	0.54	0.54	0.53	0.65	0.48	0.57	0.42	0.65	0.49
Lysine biosynthesis	0.59	0.58	0.56	0.58	0.63	0.69	0.59	0.09	0.73	0.39
Photosynthesis	0.75	0.28	0.27	0.39	0.78	0.31	0.42	0.98	0.32	0.68
Nicotinate and nicotinamide metabolism	0.47	0.49	0.50	0.50	0.45	0.48	0.42	0.76	0.44	0.59
Histidine metabolism	0.52	0.44	0.43	0.50	0.51	0.71	0.55	0.19	0.76	0.46
Pantothenate and CoA biosynthesis	0.52	0.46	0.45	0.48	0.54	0.47	0.52	0.42	0.60	0.46
RNA degradation	0.49	0.43	0.44	0.44	0.49	0.51	0.47	0.53	0.53	0.53
Pores ion channels	0.54	0.70	0.71	0.55	0.42	0.47	0.39	0.08	0.65	0.22
Signal transduction mechanisms	0.48	0.59	0.57	0.42	0.53	0.54	0.44	0.30	0.50	0.35
Glyoxylate and dicarboxylate metabolism	0.54	0.48	0.50	0.60	0.55	0.40	0.50	0.20	0.52	0.39
Galactose metabolism	0.45	0.31	0.29	0.61	0.42	0.35	0.60	0.32	0.82	0.29
Lipopolysaccharide biosynthesis proteins	0.56	0.59	0.60	0.60	0.41	0.41	0.37	0.09	0.55	0.21
Terpenoid backbone biosynthesis	0.45	0.34	0.32	0.38	0.48	0.50	0.45	0.40	0.58	0.47
Cell cycle - Caulobacter	0.43	0.36	0.37	0.41	0.42	0.43	0.45	0.52	0.45	0.54
Base excision repair	0.40	0.33	0.34	0.36	0.41	0.36	0.45	0.74	0.38	0.57
Thiamine metabolism	0.40	0.31	0.34	0.42	0.42	0.40	0.43	0.52	0.48	0.48
Valine, leucine and isoleucine degradation	0.39	0.43	0.45	0.54	0.42	0.42	0.41	0.20	0.45	0.43
Vibrio cholerae pathogenic cycle	0.45	0.96	0.91	0.45	0.35	0.43	0.23	0.07	0.20	0.08
Pentose and glucuronate interconversions	0.38	0.26	0.26	0.45	0.43	0.28	0.60	0.41	0.69	0.34
Folate biosynthesis	0.45	0.46	0.44	0.33	0.42	0.51	0.31	0.28	0.44	0.38
Drug metabolism - other enzymes	0.26	0.25	0.26	0.30	0.27	0.34	0.39	0.85	0.39	0.56
Protein kinases	0.42	0.64	0.62	0.36	0.41	0.42	0.34	0.06	0.36	0.17
Selenocompound metabolism	0.35	0.22	0.26	0.40	0.32	0.29	0.41	0.63	0.36	0.49
Fatty acid metabolism	0.41	0.48	0.50	0.59	0.42	0.30	0.36	0.09	0.31	0.25

Nucleotide excision repair	0.31	0.21	0.24	0.29	0.31	0.35	0.37	0.73	0.32	0.56
Fatty acid biosynthesis	0.44	0.36	0.35	0.41	0.46	0.43	0.40	0.06	0.50	0.28
Glycerolipid metabolism	0.39	0.30	0.28	0.35	0.35	0.28	0.36	0.62	0.23	0.45
Glutathione metabolism	0.40	0.43	0.43	0.40	0.39	0.34	0.32	0.18	0.34	0.29
Sulfur relay system	0.40	0.42	0.41	0.37	0.38	0.37	0.25	0.29	0.21	0.32
Tyrosine metabolism	0.36	0.31	0.30	0.41	0.37	0.35	0.35	0.18	0.36	0.28
Lipopolysaccharide biosynthesis	0.46	0.43	0.43	0.48	0.33	0.26	0.24	0.07	0.40	0.14
Ubiquinone and other terpenoid-quinone biosynthesis	0.42	0.49	0.48	0.36	0.39	0.26	0.25	0.06	0.34	0.12
Tryptophan metabolism	0.32	0.39	0.41	0.40	0.33	0.25	0.29	0.18	0.26	0.26
Inorganic ion transport and metabolism	0.30	0.42	0.40	0.27	0.30	0.26	0.27	0.27	0.27	0.25
Cytoskeleton proteins	0.21	0.18	0.20	0.29	0.28	0.24	0.35	0.50	0.22	0.40
Glycosyltransferases	0.33	0.30	0.29	0.33	0.33	0.30	0.35	0.05	0.43	0.16
Sulfur metabolism	0.34	0.36	0.36	0.32	0.30	0.25	0.30	0.05	0.42	0.13
Riboflavin metabolism	0.30	0.29	0.30	0.30	0.28	0.28	0.23	0.27	0.25	0.27
Lysine degradation	0.29	0.35	0.37	0.39	0.28	0.28	0.25	0.06	0.27	0.21
beta-Alanine metabolism	0.30	0.35	0.37	0.38	0.28	0.26	0.24	0.06	0.23	0.18
Cell motility and secretion	0.28	0.36	0.34	0.24	0.24	0.25	0.20	0.15	0.22	0.18
Prenyltransferases	0.31	0.21	0.20	0.23	0.32	0.27	0.26	0.04	0.36	0.18
C5-Branched dibasic acid metabolism	0.27	0.27	0.27	0.27	0.27	0.26	0.25	0.04	0.33	0.15
Other transporters	0.22	0.26	0.27	0.27	0.27	0.30	0.25	0.04	0.22	0.18
Biosynthesis of unsaturated fatty acids	0.26	0.31	0.32	0.32	0.29	0.16	0.17	0.05	0.13	0.09
Restriction enzyme	0.19	0.28	0.27	0.17	0.10	0.26	0.14	0.26	0.20	0.21
Cyanoamino acid metabolism	0.17	0.13	0.13	0.16	0.17	0.17	0.28	0.25	0.38	0.21
Streptomycin biosynthesis	0.24	0.09	0.08	0.23	0.25	0.19	0.25	0.15	0.38	0.18
RNA polymerase	0.17	0.11	0.12	0.15	0.16	0.14	0.17	0.48	0.13	0.32
Benzoate degradation	0.20	0.20	0.20	0.30	0.23	0.20	0.22	0.04	0.15	0.19

Limonene and pinene degradation	0.21	0.26	0.28	0.30	0.20	0.18	0.17	0.05	0.14	0.15
Plant-pathogen interaction	0.16	0.19	0.19	0.16	0.17	0.21	0.20	0.25	0.13	0.23
Phenylalanine metabolism	0.21	0.22	0.21	0.20	0.21	0.24	0.19	0.03	0.21	0.16
Metabolism of cofactors and vitamins	0.22	0.24	0.24	0.23	0.21	0.24	0.14	0.04	0.18	0.13
Peroxisome	0.22	0.24	0.23	0.19	0.23	0.15	0.17	0.03	0.27	0.09
Amino acid metabolism	0.18	0.14	0.12	0.17	0.21	0.38	0.14	0.03	0.23	0.21
Taurine and hypotaurine metabolism	0.15	0.17	0.17	0.15	0.15	0.13	0.16	0.37	0.13	0.24
Chloroalkane and chloroalkene degradation	0.19	0.18	0.18	0.24	0.19	0.13	0.25	0.04	0.27	0.12
Other glycan degradation	0.08	0.10	0.10	0.13	0.09	0.15	0.32	0.02	0.74	0.03
Geraniol degradation	0.17	0.25	0.27	0.31	0.17	0.12	0.15	0.05	0.09	0.11
Ascorbate and aldarate metabolism	0.20	0.23	0.19	0.16	0.10	0.12	0.14	0.25	0.14	0.16
Vitamin B6 metabolism	0.18	0.17	0.17	0.18	0.19	0.18	0.16	0.03	0.24	0.10
Sporulation	0.09	0.06	0.05	0.06	0.30	0.16	0.52	0.01	0.19	0.13
Bacterial toxins	0.15	0.23	0.22	0.16	0.11	0.14	0.12	0.14	0.09	0.11
Tuberculosis	0.18	0.15	0.15	0.13	0.18	0.14	0.13	0.14	0.13	0.14
Naphthalene degradation	0.16	0.13	0.14	0.24	0.16	0.11	0.16	0.03	0.14	0.09
Tetracycline biosynthesis	0.17	0.13	0.13	0.15	0.16	0.17	0.14	0.02	0.16	0.11
Aminobenzoate degradation	0.13	0.10	0.11	0.19	0.16	0.14	0.15	0.03	0.14	0.15
Caprolactam degradation	0.13	0.18	0.20	0.23	0.13	0.08	0.10	0.04	0.05	0.08
Biotin metabolism	0.17	0.15	0.15	0.15	0.16	0.12	0.10	0.02	0.12	0.07
Novobiocin biosynthesis	0.13	0.13	0.12	0.14	0.13	0.19	0.11	0.02	0.13	0.11
Toluene degradation	0.15	0.19	0.18	0.13	0.15	0.09	0.09	0.02	0.12	0.05
Carbohydrate metabolism	0.11	0.10	0.10	0.10	0.13	0.12	0.17	0.02	0.29	0.05
Sphingolipid metabolism	0.08	0.07	0.06	0.04	0.10	0.09	0.24	0.01	0.45	0.02
Phenylpropanoid biosynthesis	0.08	0.05	0.05	0.07	0.08	0.09	0.18	0.12	0.31	0.10
Alzheimer's disease	0.10	0.14	0.15	0.12	0.09	0.09	0.10	0.13	0.07	0.12

Polyketide sugar unit biosynthesis	0.13	0.02	0.03	0.14	0.14	0.11	0.15	0.02	0.26	0.08
Phosphatidylinositol signaling system	0.11	0.10	0.10	0.07	0.10	0.12	0.09	0.13	0.14	0.12
D-Glutamine and D-glutamate metabolism	0.12	0.10	0.10	0.13	0.13	0.14	0.10	0.02	0.13	0.08
Inositol phosphate metabolism	0.12	0.07	0.06	0.12	0.10	0.08	0.11	0.13	0.13	0.12
PPAR signaling pathway	0.12	0.08	0.07	0.10	0.13	0.07	0.10	0.13	0.13	0.10
RNA transport	0.15	0.11	0.11	0.11	0.12	0.11	0.10	0.02	0.10	0.06
Tropane, piperidine and pyridine alkaloid biosynthesis	0.11	0.10	0.09	0.10	0.11	0.16	0.09	0.02	0.12	0.09
Lipid metabolism	0.11	0.08	0.08	0.07	0.12	0.10	0.09	0.13	0.06	0.12
Huntington's disease	0.10	0.14	0.14	0.11	0.09	0.09	0.09	0.02	0.09	0.06
Vibrio cholerae infection	0.10	0.28	0.25	0.03	0.02	0.11	0.03	0.01	0.05	0.00
Lipoic acid metabolism	0.09	0.07	0.08	0.07	0.07	0.11	0.06	0.13	0.10	0.11
Lysosome	0.05	0.07	0.08	0.10	0.05	0.07	0.12	0.01	0.27	0.02
Biosynthesis of ansamycins	0.09	0.06	0.06	0.09	0.07	0.05	0.10	0.13	0.06	0.09
Nitrotoluene degradation	0.07	0.09	0.09	0.06	0.08	0.19	0.04	0.01	0.06	0.10
Glycan biosynthesis and metabolism	0.08	0.12	0.12	0.10	0.08	0.08	0.06	0.02	0.06	0.04
Drug metabolism - cytochrome P450	0.11	0.08	0.07	0.11	0.12	0.04	0.09	0.02	0.06	0.07
Polycyclic aromatic hydrocarbon degradation	0.08	0.01	0.02	0.07	0.10	0.06	0.08	0.13	0.10	0.10
Metabolism of xenobiotics by cytochrome P450	0.11	0.08	0.07	0.11	0.12	0.04	0.09	0.02	0.06	0.07
D-Alanine metabolism	0.08	0.08	0.08	0.08	0.08	0.08	0.09	0.01	0.09	0.06
Type II diabetes mellitus	0.06	0.07	0.07	0.05	0.05	0.06	0.07	0.12	0.05	0.08
Epithelial cell signaling in Helicobacter pylori infection	0.07	0.05	0.06	0.10	0.07	0.10	0.06	0.02	0.08	0.05
Ethylbenzene degradation	0.06	0.07	0.08	0.11	0.07	0.08	0.06	0.02	0.05	0.05
Biosynthesis of siderophore group nonribosomal peptides	0.08	0.18	0.16	0.02	0.04	0.07	0.03	0.00	0.06	0.01
Cell division	0.06	0.07	0.07	0.06	0.06	0.10	0.06	0.01	0.10	0.06
Glutamatergic synapse	0.07	0.05	0.05	0.07	0.09	0.07	0.08	0.01	0.06	0.05
Primary immunodeficiency	0.04	0.05	0.06	0.06	0.04	0.05	0.05	0.12	0.04	0.08

Biosynthesis and biodegradation of secondary metabolites	0.07	0.11	0.10	0.04	0.08	0.07	0.03	0.01	0.05	0.02
Isoquinoline alkaloid biosynthesis	0.06	0.05	0.05	0.06	0.06	0.11	0.04	0.01	0.07	0.06
Ribosome biogenesis in eukaryotes	0.05	0.05	0.05	0.04	0.05	0.05	0.05	0.12	0.04	0.08
Phosphonate and phosphinate metabolism	0.07	0.10	0.09	0.06	0.06	0.06	0.04	0.01	0.03	0.03
Insulin signaling pathway	0.06	0.05	0.05	0.05	0.07	0.09	0.07	0.01	0.05	0.05
Type I diabetes mellitus	0.07	0.07	0.07	0.06	0.07	0.05	0.04	0.01	0.05	0.03
Cellular antigens	0.06	0.07	0.07	0.07	0.04	0.06	0.04	0.01	0.07	0.03
Bisphenol degradation	0.06	0.05	0.05	0.07	0.07	0.03	0.06	0.01	0.05	0.03
Butirosin and neomycin biosynthesis	0.04	0.01	0.00	0.03	0.05	0.03	0.06	0.12	0.06	0.07
Arachidonic acid metabolism	0.06	0.07	0.06	0.03	0.06	0.07	0.03	0.01	0.05	0.04
Glycosphingolipid biosynthesis - globo series	0.04	0.07	0.07	0.05	0.04	0.04	0.07	0.01	0.08	0.01
Adipocytokine signaling pathway	0.06	0.05	0.04	0.04	0.05	0.04	0.04	0.01	0.11	0.01
Nucleotide metabolism	0.04	0.05	0.04	0.04	0.03	0.02	0.04	0.12	0.02	0.06
Electron transfer carriers	0.05	0.05	0.07	0.12	0.04	0.03	0.04	0.02	0.02	0.02
Ion channels	0.04	0.03	0.03	0.06	0.04	0.03	0.03	0.12	0.01	0.07
Pathways in cancer	0.05	0.05	0.05	0.05	0.05	0.05	0.05	0.01	0.05	0.04
Protein processing in endoplasmic reticulum	0.05	0.03	0.03	0.04	0.05	0.05	0.06	0.01	0.10	0.03
Parkinson's disease	0.04	0.07	0.07	0.06	0.03	0.03	0.04	0.01	0.02	0.04
Linoleic acid metabolism	0.05	0.05	0.05	0.06	0.05	0.03	0.06	0.01	0.05	0.02
Biosynthesis of vancomycin group antibiotics	0.04	0.01	0.01	0.04	0.05	0.06	0.05	0.01	0.11	0.04
Synthesis and degradation of ketone bodies	0.04	0.03	0.02	0.05	0.06	0.05	0.04	0.01	0.02	0.07
NOD-like receptor signaling pathway	0.04	0.05	0.05	0.05	0.04	0.05	0.04	0.01	0.04	0.02
Retinol metabolism	0.05	0.03	0.03	0.07	0.07	0.02	0.05	0.01	0.04	0.03
Cardiac muscle contraction	0.04	0.07	0.07	0.06	0.03	0.03	0.03	0.01	0.02	0.02
Glycosaminoglycan degradation	0.03	0.05	0.05	0.05	0.03	0.03	0.03	0.01	0.08	0.01
Dioxin degradation	0.05	0.05	0.05	0.05	0.04	0.02	0.06	0.01	0.02	0.01

Pertussis	0.03	0.07	0.06	0.02	0.03	0.04	0.03	0.00	0.06	0.01
alpha-Linolenic acid metabolism	0.04	0.05	0.06	0.08	0.04	0.02	0.03	0.01	0.01	0.02
Xylene degradation	0.04	0.05	0.05	0.05	0.03	0.02	0.06	0.01	0.02	0.01
Photosynthesis - antenna proteins	0.15	0.02	0.00	0.00	0.15	0.00	0.00	0.00	0.00	0.01
Zeatin biosynthesis	0.03	0.03	0.03	0.03	0.03	0.04	0.04	0.00	0.06	0.02
Styrene degradation	0.04	0.05	0.04	0.01	0.05	0.06	0.02	0.00	0.01	0.03
MAPK signaling pathway - yeast	0.03	0.03	0.02	0.02	0.04	0.05	0.03	0.00	0.04	0.03
Proteasome	0.03	0.02	0.02	0.02	0.04	0.04	0.03	0.00	0.04	0.02
Antigen processing and presentation	0.03	0.02	0.02	0.02	0.03	0.04	0.03	0.00	0.04	0.02
Progesterone-mediated oocyte maturation	0.03	0.02	0.02	0.02	0.03	0.04	0.03	0.00	0.04	0.02
Prostate cancer	0.03	0.02	0.02	0.02	0.03	0.04	0.03	0.00	0.04	0.02
Glycosphingolipid biosynthesis - ganglio series	0.02	0.04	0.04	0.03	0.02	0.02	0.02	0.00	0.03	0.00
Amyotrophic lateral sclerosis (ALS)	0.02	0.04	0.04	0.03	0.02	0.02	0.04	0.00	0.02	0.02
Carotenoid biosynthesis	0.06	0.01	0.00	0.01	0.07	0.01	0.03	0.00	0.03	0.02
Proximal tubule bicarbonate reclamation	0.02	0.02	0.03	0.03	0.04	0.03	0.01	0.01	0.01	0.02
Carbohydrate digestion and absorption	0.02	0.03	0.02	0.03	0.02	0.03	0.02	0.00	0.01	0.01
Penicillin and cephalosporin biosynthesis	0.04	0.01	0.01	0.03	0.04	0.00	0.03	0.00	0.01	0.02
N-Glycan biosynthesis	0.02	0.00	0.00	0.01	0.02	0.03	0.03	0.00	0.06	0.02
Atrazine degradation	0.05	0.01	0.01	0.02	0.05	0.02	0.00	0.00	0.00	0.02
Primary bile acid biosynthesis	0.01	0.02	0.03	0.03	0.01	0.01	0.03	0.01	0.01	0.01
Transcription related proteins	0.01	0.02	0.02	0.00	0.01	0.05	0.02	0.00	0.01	0.02
Non-homologous end-joining	0.03	0.02	0.02	0.02	0.03	0.01	0.01	0.00	0.01	0.01
Secondary bile acid biosynthesis	0.01	0.02	0.03	0.03	0.01	0.01	0.02	0.00	0.01	0.01
Renal cell carcinoma	0.02	0.02	0.02	0.02	0.01	0.01	0.01	0.00	0.01	0.01
beta-Lactam resistance	0.03	0.00	0.01	0.03	0.03	0.00	0.02	0.00	0.01	0.01
Ubiquitin system	0.02	0.02	0.02	0.02	0.02	0.01	0.01	0.00	0.00	0.00

Chlorocyclohexane and chlorobenzene degradation	0.02	0.00	0.00	0.01	0.02	0.01	0.02	0.00	0.03	0.02
Stilbenoid, diarylheptanoid and gingerol biosynthesis	0.02	0.00	0.00	0.01	0.02	0.02	0.00	0.00	0.00	0.02
Bladder cancer	0.01	0.02	0.02	0.02	0.01	0.01	0.01	0.00	0.00	0.00
Prion diseases	0.01	0.02	0.02	0.02	0.01	0.01	0.01	0.00	0.00	0.00
Meiosis - yeast	0.02	0.00	0.00	0.01	0.02	0.00	0.01	0.00	0.01	0.02
Steroid hormone biosynthesis	0.03	0.00	0.00	0.00	0.04	0.00	0.00	0.00	0.00	0.00
Flavonoid biosynthesis	0.02	0.00	0.00	0.01	0.01	0.02	0.00	0.00	0.00	0.01
Fluorobenzoate degradation	0.01	0.00	0.00	0.01	0.02	0.00	0.01	0.00	0.00	0.02
Chagas disease (American trypanosomiasis)	0.01	0.02	0.02	0.00	0.01	0.01	0.00	0.00	0.00	0.00
African trypanosomiasis	0.01	0.02	0.02	0.00	0.01	0.01	0.00	0.00	0.00	0.00
Bile secretion	0.01	0.02	0.02	0.00	0.01	0.01	0.00	0.00	0.00	0.00
Staphylococcus aureus infection	0.02	0.00	0.01	0.01	0.01	0.00	0.00	0.00	0.00	0.01
p53 signaling pathway	0.00	0.00	0.00	0.02	0.00	0.00	0.01	0.00	0.00	0.01
Mineral absorption	0.01	0.00	0.00	0.00	0.01	0.00	0.01	0.00	0.03	0.00
Amoebiasis	0.00	0.00	0.00	0.00	0.00	0.00	0.01	0.00	0.03	0.00
Germination	0.00	0.00	0.00	0.00	0.01	0.00	0.02	0.00	0.01	0.00
Protein digestion and absorption	0.00	0.00	0.00	0.00	0.00	0.00	0.01	0.00	0.03	0.00
Flavone and flavonol biosynthesis	0.00	0.00	0.00	0.00	0.00	0.00	0.01	0.00	0.03	0.00
Basal transcription factors	0.00	0.00	0.00	0.00	0.00	0.02	0.00	0.00	0.00	0.01
D-Arginine and D-ornithine metabolism	0.01	0.00	0.00	0.00	0.01	0.00	0.00	0.00	0.00	0.01
Apoptosis	0.00	0.00	0.00	0.01	0.00	0.00	0.01	0.00	0.00	0.01
Shigellosis	0.00	0.00	0.00	0.02	0.00	0.00	0.00	0.00	0.00	0.00
Small cell lung cancer	0.00	0.00	0.00	0.01	0.00	0.00	0.01	0.00	0.00	0.01
Colorectal cancer	0.00	0.00	0.00	0.01	0.00	0.00	0.01	0.00	0.00	0.01
Influenza A	0.00	0.00	0.00	0.01	0.00	0.00	0.01	0.00	0.00	0.01
Toxoplasmosis	0.00	0.00	0.00	0.01	0.00	0.00	0.01	0.00	0.00	0.01

Viral myocarditis	0.00	0.00	0.00	0.01	0.00	0.00	0.01	0.00	0.00	0.01
Steroid biosynthesis	0.01	0.00	0.00	0.00	0.01	0.00	0.00	0.00	0.00	0.00
Circadian rhythm - plant	0.01	0.00	0.00	0.00	0.01	0.00	0.00	0.00	0.00	0.00
Calcium signaling pathway	0.01	0.00	0.00	0.00	0.01	0.00	0.00	0.00	0.00	0.00
Ether lipid metabolism	0.00	0.00	0.00	0.00	0.00	0.00	0.00	0.00	0.00	0.00

Table S3. Summary of Bray-Curtis distance matrix (calculated with OTU table) between all the crabs

Crabs	H1	H2	H3	H4	H5	D1	D2	D3	D4	D5
H1	0.00									
H2	30.60	0.00								
H3	39.29	49.07	0.00							
H4	30.75	47.05	45.32	0.00						
H5	57.04	20.06	22.52	20.10	0.00					
D1	23.54	54.45	45.59	55.48	15.57	0.00				
D2	34.25	16.24	36.71	33.52	34.39	27.13	0.00			
D3	17.03	47.09	34.60	63.34	18.08	52.16	30.04	0.00		
D4	14.63	13.30	15.73	17.62	8.65	21.94	29.81	11.99	0.00	
D5	27.13	12.54	26.48	30.19	25.45	37.39	46.20	37.74	26.27	0.00

Table S4. Summary of Bray-Curtis distance (calculated with KEGG level 3 table) between all the crabs

Crabs	H1	H2	H3	H4	H5	D1	D2	D3	D4	D5
H1	0.00									
H2	93.52	0.00								
H3	93.50	98.57	0.00							
H4	95.15	93.47	93.96	0.00						
H5	97.82	92.62	92.71	94.46	0.00					
D1	94.43	93.27	93.35	92.93	94.66	0.00				
D2	93.89	90.67	90.83	94.05	94.46	93.18	0.00			
D3	79.09	76.48	76.82	78.31	78.56	78.34	80.47	0.00		
D4	92.54	89.50	89.70	91.40	92.67	92.87	93.79	78.27	0.00	
D5	90.07	86.68	87.01	89.50	90.10	89.62	91.64	87.22	88.18	0.00

Table S5 OTU information of each crab

OTU_ID	H1	H2	H3	H4	H5	D1	D2	D3	D4	D5
OTU2	0	21784	3279	16831	61	14443	83	16551	0	140
OTU1	5180	6861	13149	91	65	3354	155	48	1730	1
OTU7	584	3	71	332	996	35	3914	10938	0	11792
OTU6	2950	146	5395	7070	2736	555	4106	1167	151	1976
OTU5	23	1	1	307	15	9102	33	153	286	9195
OTU13	988	10	4	0	11722	0	1353	2	0	9
OTU9	73	0	0	33	19	11	10958	0	1764	1086
OTU4	5468	708	584	2457	40	184	43	358	87	14
OTU8	7380	0	0	1327	0	0	0	5	0	0
OTU3	63	32	243	95	236	189	110	18	5849	300
OTU11	11	0	7	3	0	0	222	0	6614	41

OTU10	287	42	2	0	6026	1	0	0	0	0
OTU91	44	3	4410	1	3	10	1389	0	1	1
OTU17	0	0	0	0	0	0	10	0	5137	0
OTU15	0	0	0	117	0	30	727	1	3093	86
OTU23	4	0	0	406	0	23	1587	61	306	1666
OTU20	0	0	0	88	0	966	2927	13	0	0
OTU12	1183	1	1142	154	41	310	94	27	83	652
OTU18	956	1	10	1	1932	0	3	0	0	27
OTU35	339	3	1196	6	2	41	601	12	10	78
OTU28	30	2	0	0	950	1	273	0	1	1
OTU26	379	14	2	0	765	5	18	0	0	5
OTU36	1	0	0	0	0	0	0	0	1090	0
OTU48	202	0	2	0	477	1	337	0	0	0
OTU164	471	3	6	1	406	7	1	0	0	7
OTU38	2	0	0	0	0	0	0	0	858	9
OTU42	0	0	0	0	0	15	0	0	0	841
OTU41	0	1	0	0	0	17	48	9	8	742
OTU37	3	0	0	0	0	2	103	0	605	62
OTU25	665	0	1	0	90	0	10	0	0	0
OTU40	1	0	0	0	0	0	14	0	365	242
OTU29	184	9	2	2	347	0	4	0	0	0
OTU52	166	1	0	0	318	0	3	0	0	3
OTU51	31	2	21	2	398	0	32	0	0	3
OTU44	373	0	0	5	45	2	4	1	1	39
OTU49	59	14	3	1	384	0	2	0	0	0
OTU14	203	0	61	10	16	3	88	11	4	28

OTU39	0	0	0	0	0	0	0	0	408	0
OTU193	0	0	10	141	0	44	3	122	0	1
OTU79	0	0	114	65	5	9	40	18	1	68
OTU47	116	2	2	0	136	1	6	0	0	10
OTU198	61	13	6	0	131	1	33	0	0	0
OTU182	0	22	20	3	0	189	2	9	0	0
OTU109	0	0	0	0	0	0	0	0	236	0
OTU303	137	14	3	2	66	1	1	1	0	7
OTU156	16	0	0	0	189	0	12	0	0	1
OTU43	214	0	0	0	0	0	0	0	0	0
OTU59	75	0	0	0	131	0	5	0	0	2
OTU192	0	2	1	140	0	11	0	20	0	0
OTU110	0	0	0	0	0	0	0	0	160	0
OTU151	87	2	0	1	59	0	3	0	0	1
OTU107	0	0	0	0	0	0	0	0	152	0
OTU113	0	0	0	0	0	0	0	0	150	0
OTU139	0	0	0	0	0	5	2	0	4	128
OTU82	0	0	0	79	0	51	0	0	0	0
OTU155	42	4	0	0	81	0	0	0	0	0
OTU140	0	0	0	0	1	2	0	7	0	114
OTU30	50	8	0	0	54	0	3	0	0	3
OTU236	35	1	3	1	70	0	0	0	0	4
OTU99	0	0	0	0	2	0	111	0	0	0
OTU158	48	15	0	0	37	0	0	0	0	0
OTU87	0	6	4	6	0	79	0	4	0	0
OTU116	0	0	0	0	0	0	0	0	97	0

OTU171	21	0	0	0	67	0	0	0	0	2
OTU135	3	1	1	3	5	5	8	3	0	61
OTU84	10	2	11	0	1	56	1	0	1	7
OTU200	0	36	0	0	48	1	2	0	0	0
OTU152	61	0	1	0	19	0	5	0	0	0
OTU101	3	0	0	0	1	0	28	0	29	25
OTU131	1	0	0	0	6	0	7	5	0	66
OTU104	0	2	2	13	0	2	0	66	0	0
OTU96	0	0	0	0	1	0	82	0	1	0
OTU123	0	0	0	0	0	0	6	0	75	1
OTU196	16	1	0	0	60	0	0	0	0	1
OTU120	13	0	6	0	2	0	31	0	15	7
OTU106	0	0	0	2	0	0	1	69	0	0
OTU153	12	1	0	0	58	0	0	0	0	0
OTU133	4	0	0	0	0	0	8	0	11	48
OTU102	0	0	0	2	0	0	0	67	0	2
OTU103	0	0	0	3	0	0	0	63	0	2
OTU86	0	1	3	18	0	35	0	10	0	0
OTU161	44	8	8	0	1	0	2	0	0	1
OTU83	0	0	0	0	0	1	7	0	53	2
OTU134	0	0	0	0	0	0	36	0	0	24
OTU121	0	0	0	0	0	0	0	0	60	0
OTU90	22	5	2	1	22	0	6	0	0	0
OTU130	0	0	0	0	0	0	0	0	58	0
OTU108	0	0	0	0	0	0	0	0	55	0
OTU125	0	0	0	1	0	7	0	0	44	1

OTU202	15	0	0	0	36	0	0	0	0	1
OTU180	0	1	7	10	0	27	0	2	0	0
OTU173	11	3	0	0	30	0	1	0	0	0
OTU142	0	0	0	0	0	0	0	0	43	2
OTU199	1	0	0	0	43	0	0	0	0	0
OTU176	2	16	0	0	22	0	0	0	0	0
OTU127	2	0	0	0	0	0	20	0	8	10
OTU154	28	0	0	0	11	0	0	0	0	0
OTU175	27	3	4	0	1	1	0	0	0	2
OTU46	15	0	0	0	20	0	0	0	1	1
OTU482	15	0	0	0	21	0	0	0	0	0
OTU117	0	0	0	0	0	0	9	0	25	1
OTU144	0	0	0	0	0	0	29	0	0	6
OTU126	0	0	0	0	0	0	0	0	35	0
OTU157	28	1	3	0	1	0	1	0	0	0
OTU45	10	0	1	0	20	0	0	0	0	2
OTU225	16	0	1	0	14	0	0	0	0	2
OTU141	0	0	0	0	0	0	0	0	0	32
OTU254	18	0	0	0	11	0	0	0	0	2
OTU596	16	0	0	0	14	0	0	1	0	0
OTU197	4	3	0	0	24	0	0	0	0	0
OTU118	0	0	0	0	0	0	16	0	14	1
OTU54	11	9	0	0	7	1	0	0	0	2
OTU298	8	0	0	0	20	0	0	0	0	2
OTU105	0	3	1	8	0	7	2	9	0	0
OTU268	13	0	0	0	15	0	0	0	0	1

OTU50	13	0	0	0	13	0	0	0	0	3
OTU518	12	1	0	0	13	1	0	0	0	0
OTU172	5	0	0	0	20	0	0	0	0	0
OTU94	0	0	0	0	0	0	25	0	0	0
OTU132	0	0	0	0	1	0	1	2	2	19
OTU100	0	0	0	0	0	0	8	0	14	0
OTU194	0	0	0	18	0	2	0	2	0	0
OTU195	3	0	0	0	16	0	0	0	0	2
OTU165	10	0	0	0	11	0	0	0	0	0
OTU169	16	1	0	0	3	0	0	0	0	0
OTU167	17	0	1	0	2	0	0	0	0	0
OTU201	8	1	0	0	9	0	0	0	0	1
OTU163	15	0	0	0	3	0	0	0	0	1
OTU136	0	0	0	0	0	0	1	0	0	18
OTU97	0	0	0	0	0	1	12	4	0	2
OTU227	1	14	0	0	1	1	0	0	0	0
OTU189	0	1	10	0	3	1	2	0	0	0
OTU137	0	0	0	0	0	0	1	0	1	14
OTU89	0	0	0	0	0	2	1	0	13	0
OTU168	4	0	0	0	11	0	0	0	0	0
OTU179	7	3	0	0	3	0	0	0	0	0
OTU22	0	0	0	0	3	0	0	0	0	9
OTU547	4	1	0	0	7	0	0	0	0	0
OTU93	0	0	0	0	0	0	11	0	0	1
OTU115	0	0	0	0	0	0	0	0	11	1
OTU186	1	0	8	0	0	0	2	0	0	1

OTU16	0	0	0	0	1	0	0	0	0	10
OTU19	4	0	1	0	2	0	0	0	0	4
OTU203	3	0	0	0	7	0	0	0	0	1
OTU241	5	3	0	0	1	0	0	0	0	1
OTU159	8	2	0	0	0	0	0	0	0	0
OTU414	1	0	0	0	8	0	0	0	0	1
OTU114	7	0	0	0	0	1	0	0	2	0
OTU162	0	0	10	0	0	0	0	0	0	0
OTU204	0	0	0	0	10	0	0	0	0	0
OTU337	4	0	0	0	5	0	0	0	0	0
OTU424	5	0	1	0	1	0	2	0	0	0
OTU92	0	0	0	0	0	0	1	0	8	0
OTU129	0	0	0	0	0	0	0	0	7	2
OTU170	3	0	0	0	5	0	0	0	0	0
OTU184	3	0	3	0	2	0	0	0	0	0
OTU112	0	0	0	0	0	0	0	0	8	0
OTU188	2	0	5	0	0	0	0	0	0	0
OTU174	6	0	0	0	0	0	1	0	0	0
OTU122	0	0	0	0	0	0	0	0	7	0
OTU80	0	0	1	3	0	1	0	2	0	0
OTU537	3	0	0	0	1	0	1	0	0	1
OTU388	3	0	0	0	2	0	0	0	0	1
OTU461	4	0	0	0	1	0	1	0	0	0
OTU88	0	0	0	0	0	1	5	0	0	0
OTU673	1	0	0	0	5	0	0	0	0	0
OTU230	2	0	0	0	4	0	0	0	0	0

OTU616	6	0	0	0	0	0	0	0	0	0
OTU187	0	0	5	0	0	1	0	0	0	0
OTU178	4	1	0	0	0	0	1	0	0	0
OTU98	1	0	2	0	0	1	0	0	0	2
OTU24	0	0	0	0	0	0	0	0	0	5
OTU55	1	0	0	0	0	0	0	0	0	4
OTU301	1	0	0	0	4	0	0	0	0	0
OTU583	0	0	0	0	5	0	0	0	0	0
OTU85	0	0	1	0	0	3	0	1	0	0
OTU32	0	0	0	0	0	0	0	0	0	4
OTU248	0	0	0	0	0	0	0	0	0	4
OTU286	3	1	0	0	0	0	0	0	0	0
OTU255	0	0	0	0	2	0	1	0	0	1
OTU604	2	0	0	0	2	0	0	0	0	0
OTU428	3	0	0	0	1	0	0	0	0	0
OTU53	2	0	0	0	0	0	0	0	0	1
OTU235	0	0	0	0	0	0	0	0	0	3
OTU554	2	1	0	0	0	0	0	0	0	0
OTU575	2	0	0	0	1	0	0	0	0	0
OTU333	1	2	0	0	0	0	0	0	0	0
OTU611	1	0	0	0	1	0	1	0	0	0
OTU413	2	0	0	0	1	0	0	0	0	0
OTU556	1	0	0	0	2	0	0	0	0	0
OTU143	0	0	0	0	0	0	0	0	0	3
OTU185	0	0	1	0	0	2	0	0	0	0
OTU119	0	0	0	0	0	0	0	0	3	0

OTU64	0	0	0	0	0	0	0	0	0	2
OTU27	0	0	0	0	0	0	0	0	0	2
OTU57	1	0	0	0	0	0	0	0	0	1
OTU608	1	0	1	0	0	0	0	0	0	0
OTU206	1	0	1	0	0	0	0	0	0	0
OTU208	0	0	0	0	0	0	0	0	0	2
OTU462	1	0	0	0	1	0	0	0	0	0
OTU377	0	0	0	0	2	0	0	0	0	0
OTU257	0	0	0	0	0	0	0	0	0	2
OTU220	2	0	0	0	0	0	0	0	0	0
OTU247	1	0	0	0	1	0	0	0	0	0
OTU145	0	0	0	0	0	1	0	0	1	0
OTU349	0	0	0	0	0	0	0	0	0	2
OTU477	1	1	0	0	0	0	0	0	0	0
OTU613	0	0	0	0	0	0	1	0	1	0
OTU568	0	0	0	0	2	0	0	0	0	0
OTU160	1	0	0	0	1	0	0	0	0	0
OTU124	0	0	0	0	0	0	0	0	2	0
OTU191	0	0	0	2	0	0	0	0	0	0
OTU111	0	0	0	0	0	0	0	0	2	0
OTU205	0	0	0	0	2	0	0	0	0	0
OTU138	0	0	0	0	0	0	0	0	0	2
OTU21	0	0	0	0	0	0	0	0	0	1
OTU69	0	0	0	0	0	0	0	0	0	1
OTU216	0	0	0	0	0	0	0	0	0	1
OTU290	0	0	0	0	0	0	0	0	0	1

OTU73	1	0	0	0	0	0	0	0	0	0
OTU226	0	0	0	0	0	0	0	0	0	1
OTU58	0	0	0	0	0	0	0	0	0	1
OTU218	0	0	0	0	0	0	0	0	0	1
OTU67	0	0	0	0	0	0	0	0	0	1
OTU70	0	0	0	0	0	0	0	0	0	1
OTU212	0	0	0	0	0	0	0	0	0	1
OTU432	0	1	0	0	0	0	0	0	0	0
OTU76	0	0	0	0	0	0	0	0	0	1
OTU60	0	0	0	0	0	0	0	0	0	1
OTU74	0	0	0	0	0	0	0	0	0	1
OTU34	0	0	0	0	1	0	0	0	0	0
OTU363	0	0	0	0	0	0	0	0	0	1
OTU249	0	0	0	0	0	0	0	0	0	1
OTU431	0	0	1	0	0	0	0	0	0	0
OTU443	0	0	0	0	0	0	0	0	0	1
OTU68	0	0	0	0	0	0	0	0	0	1
OTU351	0	0	0	0	0	0	0	0	0	1
OTU284	0	0	0	0	0	0	0	0	0	1
OTU528	0	0	0	0	0	0	1	0	0	0
OTU279	0	0	0	0	1	0	0	0	0	0
OTU532	0	0	0	0	0	0	0	0	0	1
OTU331	0	0	0	0	0	0	0	0	0	1
OTU280	0	0	0	0	0	0	0	0	0	1
OTU519	0	0	0	0	1	0	0	0	0	0
OTU634	1	0	0	0	0	0	0	0	0	0

OTU302	0	0	0	0	0	0	0	0	0	1
OTU525	0	0	0	0	1	0	0	0	0	0
OTU240	1	0	0	0	0	0	0	0	0	0
OTU498	0	0	0	0	0	0	0	0	0	1
OTU400	0	0	0	0	0	0	0	0	0	1
OTU320	0	0	0	0	1	0	0	0	0	0
OTU466	0	0	0	0	0	0	0	0	0	1
OTU306	0	0	0	0	1	0	0	0	0	0
OTU263	0	1	0	0	0	0	0	0	0	0
OTU481	0	0	1	0	0	0	0	0	0	0
OTU679	1	0	0	0	0	0	0	0	0	0
OTU321	1	0	0	0	0	0	0	0	0	0
OTU639	1	0	0	0	0	0	0	0	0	0
OTU467	0	0	0	0	1	0	0	0	0	0
OTU364	0	0	1	0	0	0	0	0	0	0
OTU570	1	0	0	0	0	0	0	0	0	0
OTU181	0	0	1	0	0	0	0	0	0	0
OTU183	0	0	1	0	0	0	0	0	0	0
OTU177	0	1	0	0	0	0	0	0	0	0
OTU368	0	0	0	0	0	0	0	0	0	1
OTU95	0	0	0	0	0	0	1	0	0	0
OTU128	0	0	0	0	0	0	0	0	1	0
OTU190	0	0	1	0	0	0	0	0	0	0

Table S6 Relative abundance (%) of microbial community (phylum level)

Taxon	H1	H2	H3	H4	H5	D1	D2	D3	D4	D5
Proteobacteria	63.63	96.73	92.50	88.12	53.81	64.20	30.28	60.92	9.65	15.71
Fusobacteria	18.55	2.39	1.96	8.49	3.59	0.79	1.06	1.20	0.29	0.05
Cyanobacteria	7.36	0.35	0.10	0.03	12.41	0.04	0.08	0.01	0.00	0.28
Tenericutes	3.31	0.13	5.06	1.47	4.17	0.91	15.68	37.24	19.90	41.56
Firmicutes	2.94	0.32	0.25	1.17	22.30	30.66	39.58	0.58	12.43	41.47
Bacteroidetes	1.37	0.00	0.09	0.32	0.16	3.24	10.80	0.05	46.94	0.39
Spirochaetae	0.00	0.00	0.00	0.39	0.00	0.10	2.43	0.00	10.36	0.29
Verrucomicrobia	1.32	0.05	0.01	0.00	2.63	0.02	0.06	0.00	0.00	0.02
Unclassified	0.73	0.00	0.00	0.00	0.02	0.04	0.02	0.00	0.43	0.07
Saccharibacteria	0.33	0.01	0.00	0.00	0.23	0.00	0.01	0.00	0.00	0.01
Actinobacteria	0.29	0.01	0.02	0.00	0.53	0.00	0.00	0.00	0.00	0.09
Acidobacteria	0.11	0.01	0.01	0.00	0.00	0.00	0.00	0.00	0.00	0.01
TM6_(Dependentiae)	0.06	0.00	0.00	0.00	0.12	0.00	0.00	0.00	0.00	0.00
Chloroflexi	0.01	0.00	0.01	0.00	0.01	0.00	0.00	0.00	0.00	0.01
Chlorobi	0.00	0.00	0.00	0.00	0.00	0.00	0.00	0.00	0.00	0.01
Parcubacteria	0.00	0.00	0.00	0.00	0.01	0.00	0.00	0.00	0.00	0.03

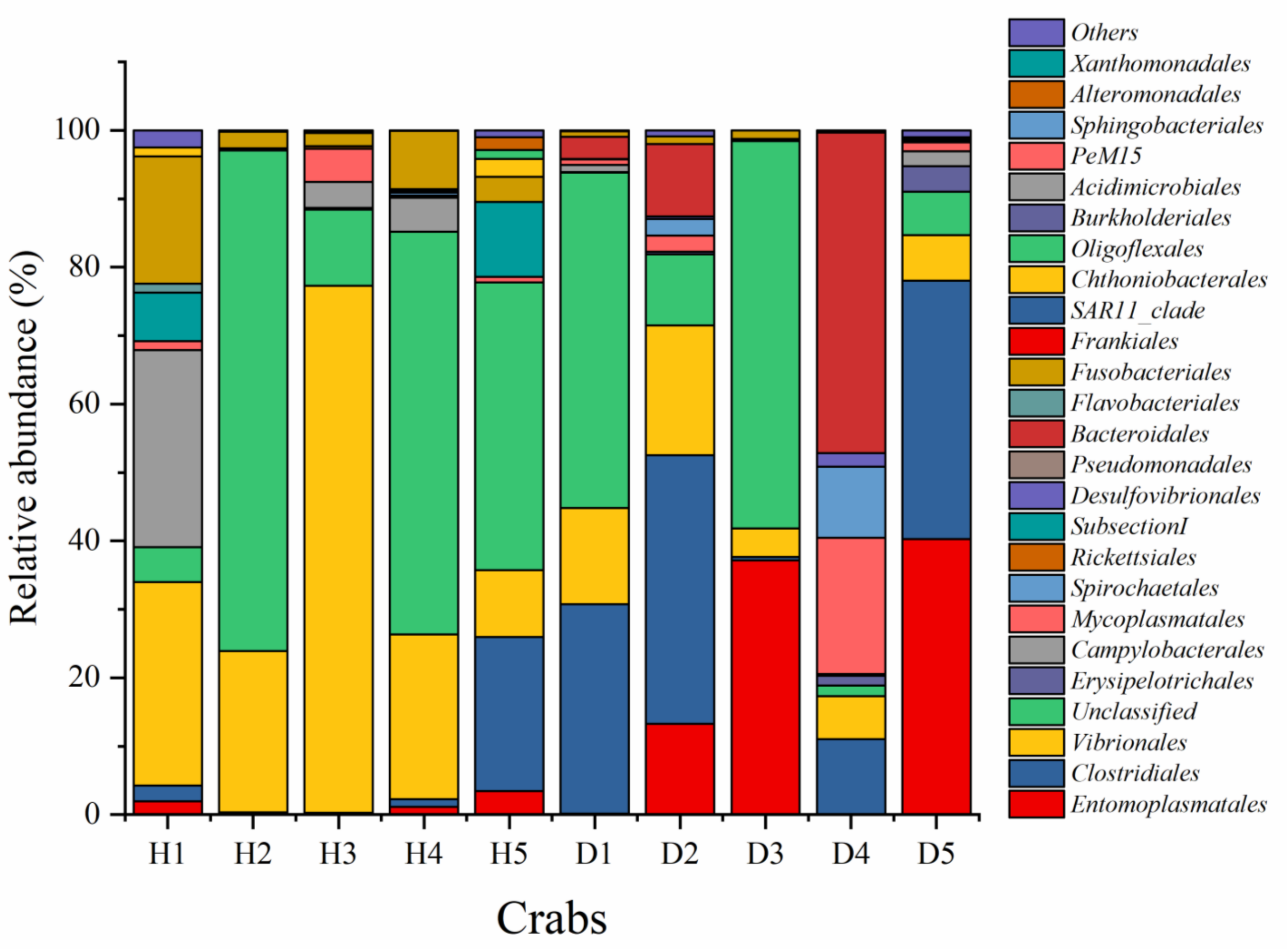

Fig. S1 Microbial community composition of the top 10 most abundant orders of each crab

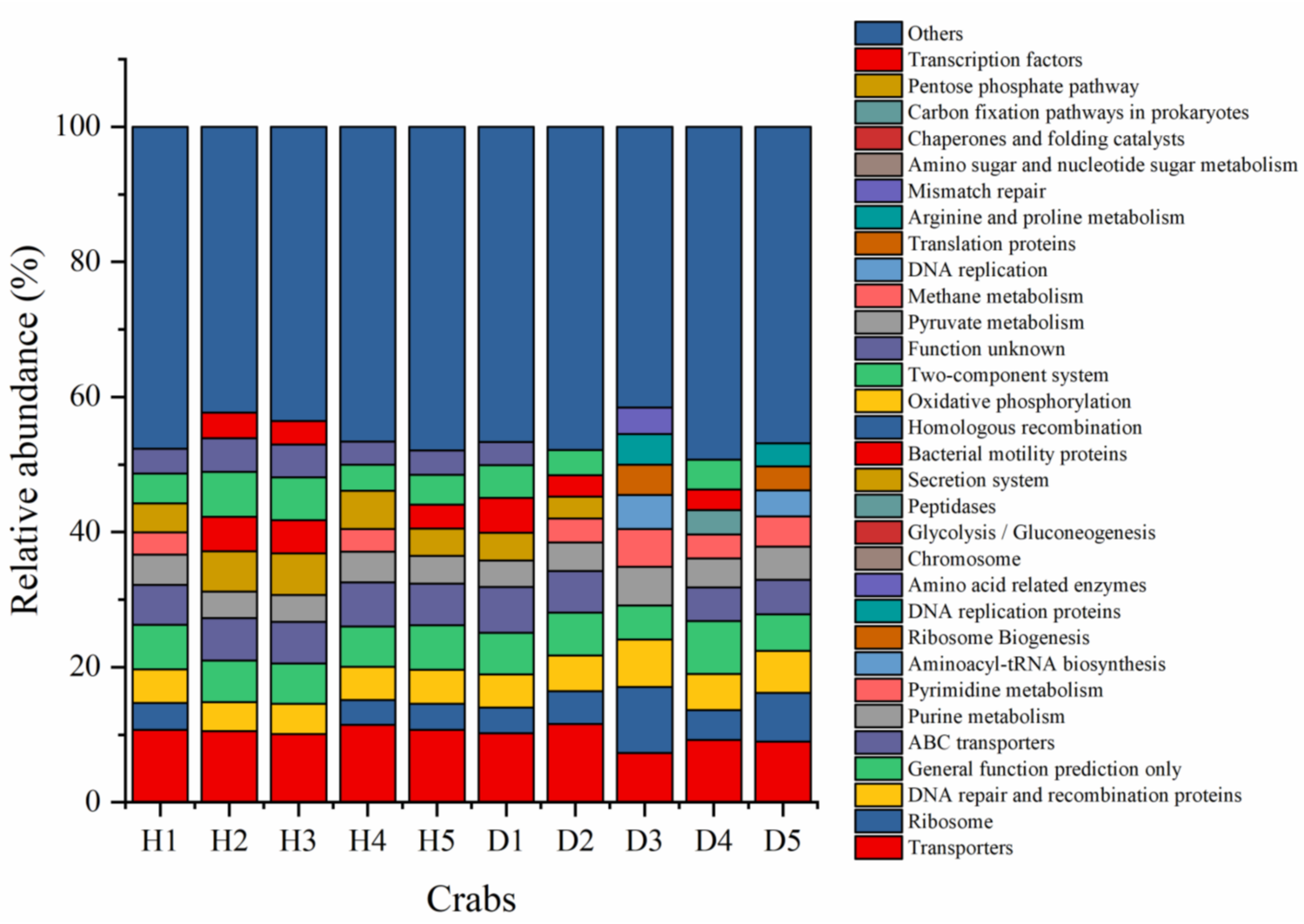

Fig. S2 Microbial functions of the top 10 most abundant KEGG level 3 categories of each crab

Table S7 Predicated functions of the intestinal microbiota that varies significantly between the healthy and diseased crabs (the relative abundance of at least one group was over than 0.5%)

CategoryL1	CategoryL2	CategoryL3	Relative abundance (%)		t value	*p* value
			Healthy	Diseased		
Environmental Information Processing	Membrane transport	Bacterial secretion system	0.90 ±0.06	0.72 ±0.04	2.537	0.035
Genes and Proteins	Protein families: signaling and cellular processes	Secretion system	2.67 ±0.24	1.57 ±0.17	3.752	0.006
Genes and Proteins	Protein families: genetic information processing	Chaperones and folding catalysts	1.07 ±0.03	0.99 ±0.02	2.356	0.046
**Genes and Proteins**	**Protein families: genetic information processing**	**Transcription machinery**	**0.66 ±0.06**	**0.91 ±0.07**	**2.771**	**0.024**
Genes and Proteins	Protein families: genetic information processing	Membrane and intracellular structural molecules	0.80 ±0.04	0.47 ±0.08	3.851	0.005
Genes and Proteins	Protein families: metabolism	Lipopolysaccharide biosynthesis proteins	0.55 ±0.04	0.32 ±0.08	2.563	0.033
**Genetic Information Processing**	**Folding, sorting and degradation**	**Protein export**	**0.49 ±0.01**	**0.67 ±0.06**	**2.858**	**0.042**
**Genetic Information Processing**	**Folding, sorting and degradation**	**RNA degradation**	**0.46 ±0.01**	**0.51 ±0.01**	**2.941**	**0.019**
Human Diseases	Infectious diseases: Bacterial	*Vibrio cholerae* pathogenic cycle	0.62 ±0.13	0.20 ±0.07	2.898	0.028
Metabolism	Carbohydrate metabolism	Butanoate metabolism	0.84 ±0.03	0.62 ±0.07	2.799	0.023
**Metabolism**	**Energy metabolism**	**Methane metabolism**	**0.94 ±0.04**	**1.07 ±0.04**	**2.610**	**0.031**
**Metabolism**	**Metabolism of cofactors and vitamins**	**One carbon pool by folate**	**0.48 ±0.02**	**0.72 ±0.08**	**3.041**	**0.035**
-	-	Function unknown	2.09 ±0.18	1.38 ±0.12	3.277	0.011
